# Similarities and Differences Between Vestibular and Cochlear Systems – A Review of Clinical and Physiological Evidence

**DOI:** 10.3389/fnins.2021.695179

**Published:** 2021-08-12

**Authors:** Ian S. Curthoys, John Wally Grant, Christopher J. Pastras, Laura Fröhlich, Daniel J. Brown

**Affiliations:** ^1^Vestibular Research Laboratory, School of Psychology, The University of Sydney, Sydney, NSW, Australia; ^2^Department of Biomedical Engineering and Mechanics, Virginia Tech, Blacksburg, VA, United States; ^3^The Menière’s Research Laboratory, Sydney Medical School, The University of Sydney, Sydney, NSW, Australia; ^4^Department of Otorhinolaryngology, Head and Neck Surgery, Martin Luther University Halle-Wittenberg, Halle, Germany; ^5^School of Pharmacy and Biomedical Sciences, Curtin University, Bentley, WA, Australia

**Keywords:** vestibular, otolith, labyrinth, vemp, semicircular canal, saccular, utricular

## Abstract

The evoked response to repeated brief stimuli, such as clicks or short tone bursts, is used for clinical evaluation of the function of both the auditory and vestibular systems. One auditory response is a neural potential — the Auditory Brainstem Response (ABR) — recorded by surface electrodes on the head. The clinical analogue for testing the otolithic response to abrupt sounds and vibration is the myogenic potential recorded from tensed muscles — the vestibular evoked myogenic potential (VEMP). VEMPs have provided clinicians with a long sought-after tool — a simple, clinically realistic indicator of the function of each of the 4 otolithic sensory regions. We review the basic neural evidence for VEMPs and discuss the similarities and differences between otolithic and cochlear receptors and afferents. VEMPs are probably initiated by sound or vibration selectively activating afferent neurons with irregular resting discharge originating from the unique type I receptors at a specialized region of the otolithic maculae (the striola). We review how changes in VEMP responses indicate the functional state of peripheral vestibular function and the likely transduction mechanisms allowing otolithic receptors and afferents to trigger such very short latency responses. In section “ELECTROPHYSIOLOGY” we show how cochlear and vestibular receptors and afferents have many similar electrophysiological characteristics [e.g., both generate microphonics, summating potentials, and compound action potentials (the vestibular evoked potential, VsEP)]. Recent electrophysiological evidence shows that the hydrodynamic changes in the labyrinth caused by increased fluid volume (endolymphatic hydrops), change the responses of utricular receptors and afferents in a way which mimics the changes in vestibular function attributed to endolymphatic hydrops in human patients. In section “MECHANICS OF OTOLITHS IN VEMPS TESTING” we show how the major VEMP results (latency and frequency response) follow from modeling the physical characteristics of the macula (dimensions, stiffness etc.). In particular, the structure and mechanical operation of the utricular macula explains the very fast response of the type I receptors and irregular afferents which is the very basis of VEMPs and these structural changes of the macula in Menière’s Disease (MD) predict the upward shift of VEMP tuning in these patients.

## Introduction

Similar goals have driven both auditory and vestibular research – the need for clinical tests to identify disorders of the sensory system. For an excellent reference for much of the basic vestibular neurophysiology discussed here see Goldberg et al. (2012). In the case of hearing, the audiogram has been a key test and vestibular research started, similarly, with a strong emphasis on a “vestibulogram” or “cupulogram”- measuring vestibular thresholds. This was mainly from investigators in the Netherlands around 1950 e.g. Groen and Jongkees (1948). Paralleling the results of those threshold studies were basic science studies such as the development and elaboration of the Steinhausen torsion pendulum model of semicircular canal operation (Van Egmond et al., 1949; Groen, 1957; Straka et al., 2021), measurement of neural responses and modeling of the peripheral semicircular canal (and otolith) mechanisms determining the thresholds (von Bekesy, 1955) and the first mechanical model of otolith function (de Vries, 1950). However, the use of psychophysical thresholds to vestibular stimulation did not prove to be as useful in clinical assessment of vestibular function as it has been for audition. Vestibular thresholds are difficult to measure, of limited reliability and it is costly and clinically impractical to deliver angular and linear accelerations safely to a whole person to determine their thresholds. So vestibular threshold studies have largely remained in the lab (Clark, 1967; Karmali et al., 2016; Kobel et al., 2021) although they have contributed to models of vestibular mechanics.

**FIGURE 1:**
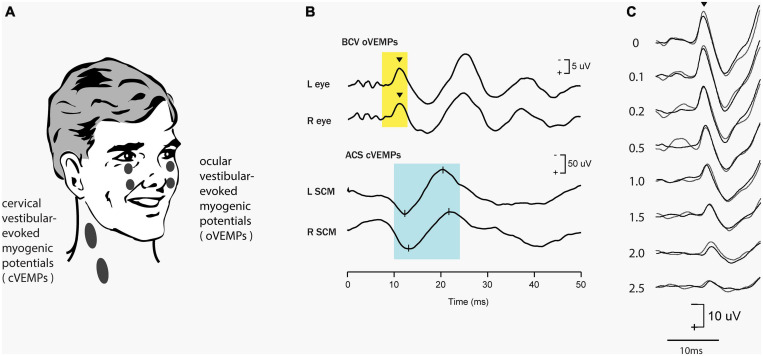
Vestibular evoked myogenic potentials (VEMPs). There are a host of VEMPs since vestibular input projects indirectly to many muscle groups. The two VEMPs which have received the most attention are cervical VEMPs (cVEMPs) and ocular VEMPs (oVEMPs). cVEMPs are recorded by surface EMG electrodes over the tensed sternocleidomastoid muscles (SCM) (Colebatch and Halmagyi, 1992; Colebatch et al., 1994; Adams et al., 2010). The cVEMP consists of a short-latency (13 ms from onset to peak) positive (i.e., inhibitory) EMG potential in response to high-intensity ACS or BCV (Halmagyi et al., 1995). Ocular VEMPs (oVEMPs) consist of a small (5–10 μV) negative (i.e., excitatory) potentials recorded by surface electrodes on the skin beneath the eyes from the inferior oblique in response to BCV or ACS (Rosengren et al., 2005; Iwasaki et al., 2007). To record the oVEMP the subject must be looking up. **(A)** Electrode placement for oVEMPs and cVEMPs; the ground electrode (not shown) is typically on the chin or sternum. **(B)** Typical oVEMP and cVEMP records for a healthy subject in response to bilaterally equal amplitude stimuli: the magnitude of the n10 response is approximately equal beneath both eyes for the oVEMP, and, similarly, the magnitude of the p13-n23 response is approximately equal in both SCMs for the cVEMP. **(C)** For stimuli in the one subject, increasing the rise time of the 500 Hz stimulus reduces the amplitude of the oVEMP (Burgess et al., 2013). The number next to each record shows the rise time in milliseconds. Reproduced from Frontiers.

Clinical auditory testing progressively turned to objective tests – where clicks or short tone bursts elicit the auditory brainstem response (ABR) which is a neural response recorded by surface electrodes on the head and is used to evaluate dysfunction in the auditory system (Rowe, 1981; Eggermont, 2017; Drexl et al., 2018). The ABR is a short-latency response generated by neural structures in the ascending hearing pathway of the auditory brainstem, including a post-stimulus interval of 1 to 12 ms. The evaluation of latencies and amplitudes of peaks in the ABR waveform allows for the assessment of dysfunction in the auditory system. In parallel fashion vestibular investigators have turned to measuring objective vestibular tests – vestibular evoked responses—with the prime evoked response being the vestibular evoked myogenic potential (VEMP). This is a short latency myogenic potential evoked by air-conducted sound (ACS) or bone-conducted vibration (BCV) (mainly clicks or short (7 ms) tone bursts of 500 Hz) either in tensed sternocleidomastoid (SCM) neck muscles [the cervical VEMP (cVEMP) or in stretched inferior oblique (IO) eye muscles (the ocular VEMP (oVEMP)]. For a history of VEMPs see Colebatch and Halmagyi (1992), Colebatch et al. (1994), Curthoys et al. (2018), Rosengren and Colebatch (2018) (see Figure 1). The amplitude of the oVEMP is largest for short rise time stimuli (Burgess et al., 2013) as analogous to the wave I of the ABR (Finneran et al., 2018)– pointing to the synchronous activation of primary vestibular afferents in this case as having a major role in the generation of the evoked response (Goldstein and Kiang, 1958).

### The VEMP

**FIGURE 2:**
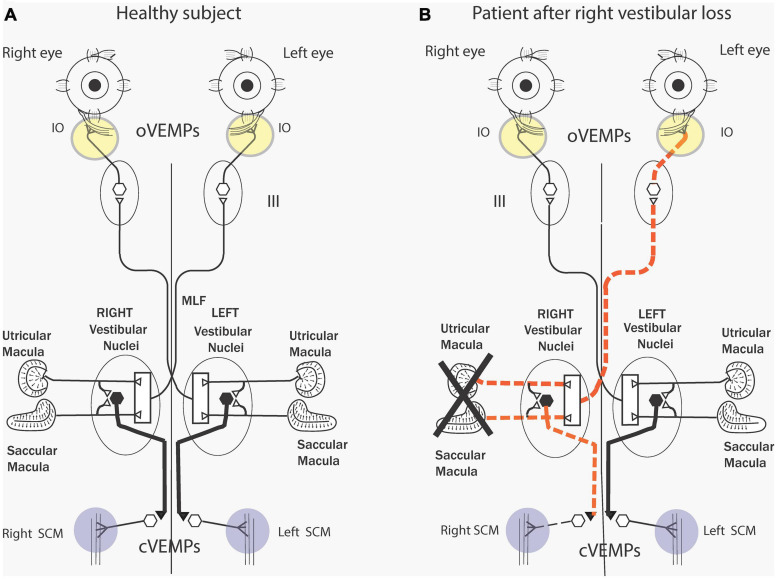
**(A)** Simplified schematic diagram of some of the known neural vestibulo-ocular and vestibulo-collic projections that underlie the oVEMP and cVEMP myogenic responses. The Figure is based on known anatomical projections (Uchino and Kushiro, 2011) and on physiological results that high-frequency electrical stimulation of the utricular nerve results in the activation of the contralateral inferior oblique (IO) (Suzuki et al., 1969). Electrodes beneath the eyes record the oVEMP as the person looks up. Afferents from the saccular and utricular macula project to the vestibular nuclei, but the exact termination of these afferents with the nucleus is still unclear so this figure represents the present uncertainty about the exact neural connections of these afferents within the nuclei as an open box. The projections of the saccular macula in the inferior vestibular nerve, synapsing on an inhibitory neuron in the vestibular nucleus (*thick black lines*), projecting to spinal motoneurons controlling the sternocleidomastoid muscle (SCM), are established (Uchino and Kushiro, 2011). Electrodes over contracted SCM muscles record the cVEMP. Unilateral vestibular loss **(B)** has been shown to result in reduced or absent contralateral oVEMP n10 and a reduced or absent ipsilateral cVEMP. Reproduced from Curthoys et al. (2011a) with permission from Wiley.

As we discuss in detail below an abrupt sound or vibration stimulus activates, within one millisecond, a small subgroup of otolithic receptors and afferents with irregular resting discharge – originating from the central striolar area of the otolithic sensory regions (Curthoys et al., 2006; Curthoys and Vulovic, 2011). The afferents from the utricular macula project via the vestibular nuclei and oculomotor nuclei to the contralateral IO (Suzuki et al., 1969) causing short latency (7 ms) myogenic potentials in these ocular muscles which can be recorded by surface electrodes above the IO eye muscles as the patient looks up (Figure 2). This response is identified as the ocular VEMP (oVEMP) and is predominantly of utricular origin (Curthoys, 2010). The anatomical projections from the vestibular nuclei to the eye muscles are partially known (Uchino and Kushiro, 2011) but recordings from single motor units in human eye muscles conclusively show that clicks generate short latency excitation in inferior oblique eye muscles (Rosengren et al., 2011; Weber et al., 2012). Afferents from the saccular macula project to the ipsilateral sternocleidomastoid (SCM) causing short latency inhibitory myogenic potentials in these muscles, again recordable by surface electrodes above the tensed SCM. This response is predominantly saccular and is identified as the cervical VEMP (cVEMP). There are similar short latency inhibitory single motor unit responses in human cervical muscle units (Rosengren et al., 2015) in response to BCV.

These data serve as the physiological basis for the clinical use of VEMPs to indicate the functional status of the peripheral otolithic receptors in each labyrinth. So, the one stimulus generates responses which probe the function of both the utricular and the saccular maculae (Curthoys, 2010) and unilateral otolithic loss shows the reduced myogenic response from the affected ear (Figure 2B). Reviews give extensive information about the history, the detailed physiology and how to perform these VEMP tests clinically (MacDougall et al., 2015; Dlugaiczyk, 2017; Rosengren and Colebatch, 2018; van de Berg et al., 2018; Rosengren et al., 2019). VEMPs are now being used as a routine indicator of the operation of otolithic receptors in patients with probable vestibular neuritis or even with intracochlear schwannoma (Frohlich et al., 2020) and also to pinpoint probable central neural deficits (Oh et al., 2016). Hence, just as the ABR is used to assess peripheral (and central) auditory dysfunction, the VEMP is being used clinically to probe peripheral (and central) vestibular function.

Clicks or short tone bursts of sound or vibration are used to assess the function of both the vestibular and the auditory system because physiological evidence has shown that some vestibular receptors and afferents are activated at very short latency by these stimuli (Curthoys et al., 2006, 2014; Curthoys and Vulovic, 2011; Pastras et al., 2018). There has been debate whether VEMPs contain an auditory contribution in human patients. However, a wealth of clinical and physiological evidence conclusively shows the vestibular (and specifically otolithic) origin of VEMPs (for reviews see Rosengren and Colebatch, 2005; Iwasaki et al., 2008; Curthoys et al., 2011a; Curthoys, 2020; Curthoys and Dlugaiczyk, 2020).

The major clinical VEMP results are: (1) unilateral vestibular loss (due to surgery for vestibular schwannoma removal or vestibular neuritis) causes loss of ipsilateral cVEMPs and contralateral oVEMPs corresponding to the neural projection pattern shown in Figure 2 (Colebatch and Halmagyi, 1992; Iwasaki et al., 2007; Figure 2). (2) Thinning of the bony wall of the anterior semicircular canal [known as semicircular canal dehiscence (SCD)] changes the mechanical operation of the labyrinth and affects both cochlear and vestibular receptors (Rosowski et al., 2004; Curthoys, 2017; Iversen and Rabbitt, 2017; Iversen et al., 2018; Dlugaiczyk et al., 2019; Rabbitt, 2019). SCD enhances the neural response of otolith neurons and activates previously unresponsive semicircular canal neurons with irregular resting discharge to sound and vibration and so causes enhanced VEMP responses (Colebatch et al., 1998; Manzari et al., 2013; Curthoys, 2017; Dlugaiczyk et al., 2019).

**FIGURE 3:**
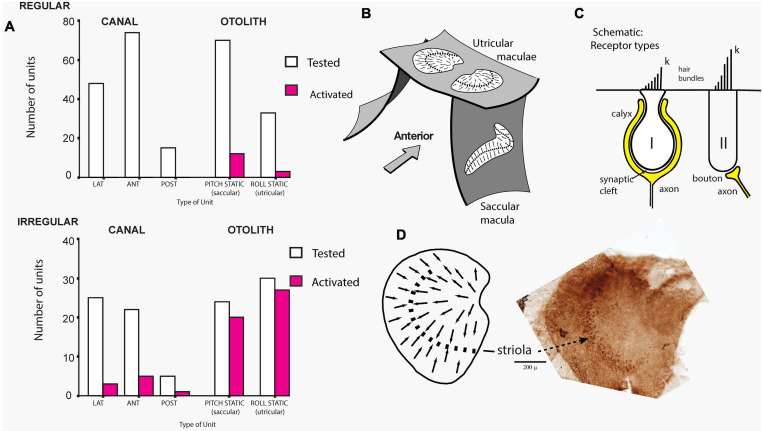
**(A)** Proportions of primary vestibular afferent neurons tested (open bars) and activated (filled bars) by bone-conducted vibration (BCV) stimuli from each vestibular sensory region. Top chart - Regular neurons, bottom chart irregular neurons. Lat, horizontal canal; Ant, anterior canal; Post, posterior canal. The otolith neurons are divided into Pitch static and Roll static. The results show that only a very small proportion of semicircular canal neurons, or regular otolithic neurons, were activated. However, a large proportion of otolithic irregular neurons were activated. Reproduced, from Curthoys et al. (2006) with permission of Springer. **(B)** Schematic representation of the plates of otolithic receptors (the utricular and saccular maculae) of the inner ear. The arrows show the preferred polarizations of hair cell receptors across the maculae. The dashed lines are lines of polarity reversal, where the preferred polarizations of receptors reverse. The striola refers to a thin band of receptors on either side of the line of polarity reversal. **(C)** Schematic representation of type I and type II receptor hair cells and their calyx and bouton afferent terminals. The longest cilium, the kinocilium (k), defines the preferred direction of cell polarization (shown by small arrows in **B** and **D**). The type I receptor is enveloped by the calyx afferent ending. **(D)** Schematic representation of a dorsal view of the whole guinea pig utricular macula. Adjacent to this schematic is a dorsal view of a guinea pig utricular macula treated by calretinin—the band of cells comprising the striola is clearly visible. Reproduced from Curthoys et al. (2012), with permission from Elsevier.

The cause of that enhanced response was demonstrated in guinea pigs where single canal or otolith neurons were recorded continuously while the dehiscence was carried out, and it was found that after SCD, ACS and BCV enhanced the activation of the irregular otolithic afferents, but also activated irregular semicircular canal neurons, which had been unresponsive to these stimuli prior to the SCD (Figure 3). Both canal and otolith neurons showed phase locking up to very high stimulus frequencies (even beyond 3000 Hz). The contribution of canal neurons was shown by the fact that canal neurons which were activated after SCD (which had been unresponsive prior to the SCD) (Figure 3) again became unresponsive after the SCD was resealed. After SCD otolith neurons showed a lower threshold and enhanced response to higher frequencies in contrast to their more limited high frequency response prior to SCD (Curthoys et al., 2006; Curthoys and Vulovic, 2011; Curthoys, 2017; Curthoys and Dlugaiczyk, 2020). In corresponding fashion human patients with SCD show enhanced amplitude VEMPs to 500 Hz stimuli but also show clearVEMPs to very high frequency “vestibular” stimuli (4000 Hz) whereas healthy subjects do not have VEMPs to such high vestibular frequencies, thus making the VEMP response to 4000 Hz an excellent, simple, specific indicator of human SCD (Manzari et al., 2013; Curthoys and Manzari, 2020; Noij and Rauch, 2020). Note, that “high frequency” with regards to vestibular stimulation (and the VEMP) refers to frequencies greater than about 500 Hz, which is much lower than what is considered high frequency in the cochlea.

### Sound and Vibration in the Cochlea and Otoliths

Prior to discussing the various sensitivities of the cochlea and vestibular system to ACS or BCV stimuli of various frequencies, it is worth noting that it is not a straightforward task to compare a system’s sensitivity to these different modes of stimulation. That is, whilst colloquially we may state that the vestibular system is more sensitive to vibration than sound, and the cochlea more sensitive to sound than vibration, in isolation we cannot make this comparison. That is, ACS and BCV level are usually measured in completely different, non-comparable units. The level of an ACS stimulus is relatively straightforward to calibrate, being the sound level in the ear canal, in units of dB SPL (or dB nHL for stimuli of short duration with reference to hearing levels). Calibrating the level of a BCV stimulus requires significantly more consideration, as it should ideally be provided in units of acceleration of the skull (or some permutation of such, e.g., vibratory force level dB VFL, as it is done for audiometric applications). The measurement depends upon where the BCV stimulator is placed, and where the acceleration is measured (Govender et al., 2015; Govender and Colebatch, 2017; Govender and Colebatch, 2018) (not to mention a likely large variability in physical properties of the skull across individuals which will influence the vibration in the otoliths, particularly for transient stimuli).

That said, in the clinical setting, for evoking responses such as the VEMP or ABR, the BCV device is likely to be something like a RadioEar B81 (or B71) audiometry grade transducer, placed over the mastoid bone. Whilst there is unlikely to be any effort to measure the acceleration of the skull, if the same “voltage source” (i.e., from the audiometer device) is used to drive both the calibrated headphones (or earphones) for ACS stimulation, and the BCV transducer, then the clinician may be tempted to draw some approximations about the “relative” sensitivity of responses such as the ABR and VEMP to BCV or ACS in units of dB SPL, dB VFL, or dB nHL. But even then, it must be understood that comparing the voltage drive to a headphone or bone-conductor required to evoke an ABR (or VEMP) at threshold, does not tell you about the relative sensitivity of the cochlea (or vestibular system) to ACS or BCV. It merely tells you about the relative efficiency of that headphone or bone conductor to produce stimulation within the ear, for a given voltage drive. However, we can compare the relative sensitivity of the cochlea vs. otoliths to a given stimulus (either a BCV or an ACS stimulus). To evoke a VEMP at threshold, using say a 500 Hz ACS stimulus, requires roughly 60 to 70 dB higher sound level than that which is required to evoke an ABR using the same stimulus (Gorga et al., 1993; Rosengren and Colebatch, 2018). Conversely, to evoke a VEMP using say a 500 Hz BCV stimulus (a B81 bone conductor placed on the mastoid), requires approximately 40 to 60 dB higher vibratory force level compared to evoking an ABR (Hakansson et al., 2018; Mueller et al., 2020; Frohlich et al., 2021). If we reference BCV level relative to ABR thresholds (or hearing thresholds), in terms of dB nHL, it could potentially be argued that the vestibular system is about 20 to 30 dB more sensitive to BCV than ACS, for this setup, using these devices (Groen and Jongkees, 1948). This does not suggest that the otolith is 20 to 30 dB more sensitive for BCV than ACS, as this will depend upon the specifics of the BCV stimulus and the comparison is based on terms of hearing levels (dB nHL), i.e., audiometric units specifying sensitivity relative to the cochlea.

### VEMPs Frequency Response and Tuning Shift

In healthy subjects the optimum frequency for evoking VEMPs to short tone burst stimuli with a 2 ms rise time (either ACS or BCV) is around 500 Hz, with usually a clear decrease in VEMP amplitude in healthy subjects in response to 1000 Hz (Lin et al., 2006; Timmer et al., 2006; Piker et al., 2013; Singh and Barman, 2016a,b; Singh and Firdose, 2018; Noij et al., 2019). The VEMP frequency response depends on many factors but of major importance are the physical characteristics of the macula (thickness, stiffness etc.) and the attachment of the otolithic receptors to the overlying otolithic membrane. The receptors at the specialized region of the macula, the striola (see Figures 3B,C), have short stiff hair bundles (Spoon and Grant, 2011, 2013) with tenuous attachment to the overlying otolithic membrane (Lindeman, 1969) and so are similar to cochlear inner hair cells which have minimal tenuous attachment to the tectorial membrane (Dallos, 1992; Hakizimana and Fridberger, 2021). In contrast the receptor hair cells of the extrastriolar regions of the otolithic maculae have long hair bundles which extend far up into the otolithic membrane and appear to have much tighter attachment to the otolithic membrane than the striolar receptors (Spoon et al., 2011).

In patients with Menière’s Disease (MD) the optimum stimulus frequency for VEMPs is around 1000 Hz with a smaller response to 500 Hz. This is the opposite of the response preference in healthy subjects where 500 Hz causes a larger response than 1000 Hz. So, the ratio of VEMP amplitude at 500 Hz to VEMP amplitude at 1000 Hz is becoming recognized as an indicator of Menière’s Disease – the “upward shift” of tuning in MD patients (Lin et al., 2006; Timmer et al., 2006; Piker et al., 2013; Noij et al., 2019). The major question is why patients with MD show this upward tuning shift and below (Section “MECHANICS OF OTOLITHS IN VEMPS TESTING”) we show how the change in the thickness of the otolithic membrane (and so its stiffness) during MD (Calzada et al., 2012; Ishiyama et al., 2015) changes the mechanical tuning of the otolithic macula and predicts such an upward tuning shift of VEMPs.

### Cochlear and Vestibular Hair Cells and Afferents

Both the cochlea and vestibular systems have two distinct types of receptor hair cells which play unique roles in mechanoelectrical transduction and sensory processing. The cochlea hosts inner hair cells (IHCs) and outer hair cells (OHCs), whereas the vestibular sensory regions exhibit a similar dichotomy in the type I and type II hair cells (see Goldberg, 2000; Eatock and Songer, 2011; Goldberg et al., 2012) for full details (Figure 3). These cochlear and vestibular receptor cells are broadly similar in structure and function and modulate gating current via displacements of their stretch sensitive tip-links at the apices of the hair bundles. In the cochlea, OHCs are biological ‘motors’ which actively vibrate to overcome negative damping, likely driven by the electromotile protein, prestin. By contrast, IHCs are primarily ‘sensors’ which relay auditory information to the CNS, via spiral ganglion fibres ∼95% of which contact IHCs and only about ∼5% contact OHCs. Cochlear afferent fibres exhibit a range of characteristics, with essentially two sub-types: (1) those with a high spontaneous firing and low threshold (to sound); and (2) those with a low spontaneous rate and high threshold (Heil and Peterson, 2015, 2017). In the vestibular system, there is no motor vs sensor dichotomy comparable to the cochlear OHCs vs IHCs, but rather different receptor-afferent systems with distinct sensitivities. Type II hair cells have a considerably depolarized Resting Membrane Potential (RMP), at around −50 mV, determined by several inwardly and outwardly rectifying K+ currents (Holt and Eatock, 1995; Eatock and Songer, 2011). Type II hair cells synapse on bouton afferent fibres, which have tonic response dynamics, high thresholds, and low sensitivity to linear or angular forces. By contrast, type I hair cells have a relatively hyperpolarized RMP at −80 mV, dominated by a large, slowly activating K+ current (I_K,L_) (Correia and Lang, 1990), that synapse on calyx afferent fibres, which have phasic response dynamics, low thresholds and high sensitivity to linear and angular forces (Eatock and Songer, 2011). Interestingly, dimorphic afferent fibres terminate on both types of hair cells, and generally have response properties akin to calyx units (Fernandez et al., 1990; Goldberg et al., 1990).

### Vestibular Primary Afferent Neurons – Spontaneous Activity

**FIGURE 4:**
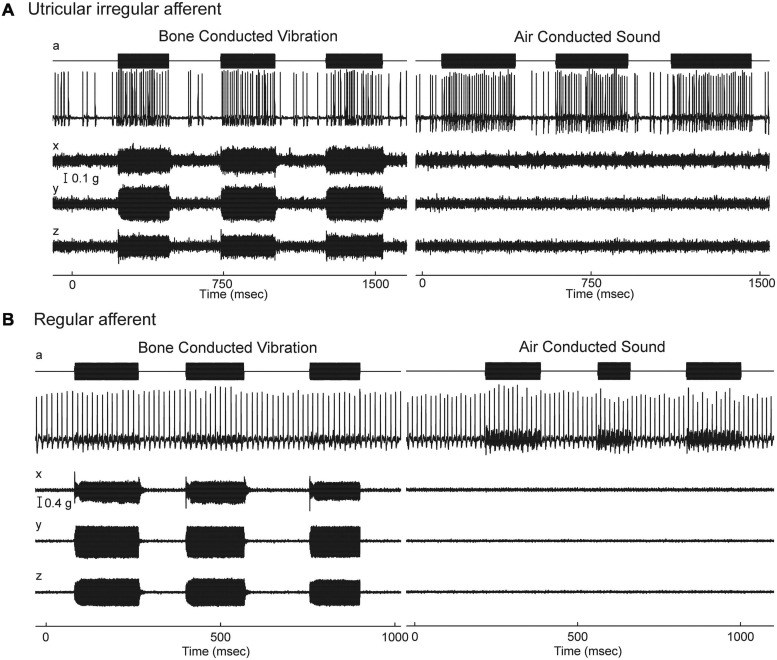
Resting discharge pattern and response to stimulation of an irregular and a regular afferent. **(A)** Time series of the one irregular otolith neuron during stimulation by 500 Hz bone-conducted vibration (BCV) and air-conducted sound (ACS). The top trace (a) shows the command voltage, indicating when the stimulus is on. The second trace shows the action potentials by extracellular recording. The three bottom traces (*x, y, z*) show the triaxial accelerometer recording of the stimulus. The neuron is clearly activated by both BCV and ACS. **(B)** Time series of a regular semicircular canal neuron during stimulation by 500 Hz BCV and ACS as above. The regular discharge is seen before the stimulus onset. The stimuli have a far greater amplitude than in panel **(A)**, but there is no evidence of activation of this regular neuron by these strong stimuli. Reproduced from Curthoys and Vulovic (2011) with permission of Springer.

**FIGURE 5:**
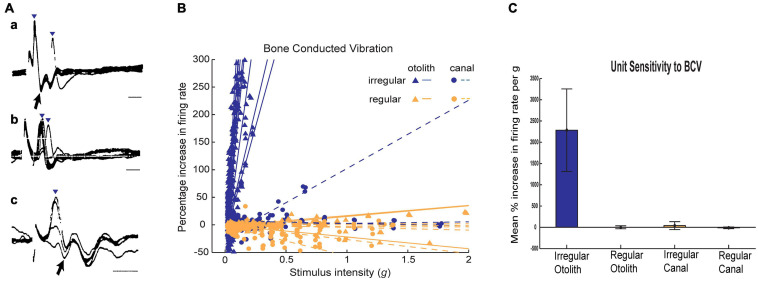
**(A)** Irregular otolithic primary afferents are evoked by air conducted clicks at very short latencies (from Murofushi et al., 1995). Three examples of air conducted click-evoked action potentials (identified by inverted triangles) in three primary otolithic neurons. Superimposed recordings of responses to 5–10 clicks. Latencies from the onset of the click to the foot of the action potential were 0.5 ms (a,c) and 1.0 ms (b). b and c show responses at threshold-straddling intensities, so the action potential is not evoked on every presentation. Note that these action potentials (a, c) occur at such short latencies they precede the N1 wave of the acoustically evoked cochlear action potential (arrows). Time scales 1 ms. Reproduced from Murofushi et al. (1995) with Permission of Springer **(B)** Examples of sensitivity plots of neurons to BCV showing the high sensitivity of irregular neurons to increasing BCV stimulus strength as opposed to regular afferents. Each point shows the increase in firing rate as the percentage of baseline firing rate during a single stimulus presentation. Each *line* is the best fit calculation of the responses for one neuron (triangles - otolith neurons, circles-semicircular canal neurons; irregular afferents are blue and regular afferents are orange. The stimulus intensity is calculated in *g*, and is the root mean square of three axes as recorded by the skull-mounted triaxial accelerometer. Canal neurons and regular otolithic afferents are not activated by high stimulus levels. In contrast irregular otolithic afferents are activated at very low intensities and have a very steep increase in firing as intensity is increased. **(C)** Average sensitivities for neurons to BCV. The slopes of the best fitting lines in **(B)** are averaged for each class of neuron, and the average slope and 95% confidence intervals. The unit of sensitivity in this plot is per cent increase in firing rate per *g* above the resting discharge rate. The high sensitivity of otolith irregular neurons and the absence of response of otolith regular and semicircular canal neurons is clear. Reproduced from Curthoys and Vulovic (2011) with permission of Springer.

Given the gross anatomical similarity of vestibular receptor hair cells and cochlear hair cells, it is surprising that the resting activity of afferent neurons in the two systems is so different (Walsh et al., 1972; McCue and Guinan, 1995; Heil et al., 2007; Heil and Peterson, 2015; Curthoys et al., 2016). Cochlear afferents, almost all of which arise from IHCs, have resting discharge which is irregular (Heil and Peterson, 2015, 2017). In contrast the regularity of resting discharge of primary vestibular afferents is a continuum (Goldberg, 2000) which, partly for convenience, has been divided into two main categories - some with very regular resting rates which have been shown to originate from extrastriolar dimorphic and bouton afferents synapsing on type II receptors (Fernandez et al., 1990). Other neurons with irregular resting discharge, have been shown to originate predominantly from calyx endings on type I receptors at the striola (Fernandez et al., 1990; Curthoys et al., 2012). In the vestibular system the regularity of resting discharge is associated with functional differences: recordings of single primary otolithic afferent neurons in guinea pigs, cats, squirrel monkeys have shown that sounds and vibrations are effective in activating the otolithic afferents with irregular resting discharge whereas these same stimuli are ineffective at activating afferents with regular resting discharge (see Figures 4, 5), So the ACS and BCV stimuli used to generate VEMPs in patients in the clinic, selectively activate irregular dimorphic and calyx afferents synapsing on receptors at the striola of the otolithic macula where the afferents form unique calyx synapses on the amphora shaped type I receptors (Curthoys and Vulovic, 2011; Curthoys et al., 2012, 2019a; Curthoys, 2017).

Eatock and Kalluri suggest that the different resting discharge patterns of vestibular afferents probably reflect differences in the operation of channels on the cell membrane (Eatock et al., 2008; Kalluri et al., 2010). A recent study in the guinea pig confirmed that many irregular vestibular afferents have very low (or even zero) resting discharge (Yagi et al., 1977; Curthoys et al., 2016), which is comparatively rare in auditory afferent neurons (Walsh et al., 1972). The very low resting rate of some irregular vestibular afferents likely reflects the unique physiology of the vestibular calyx synapse on type I receptors at the striola (Lysakowski et al., 2011; Lim et al., 2011; Contini et al., 2017; Contini et al., 2020).

### Vestibular Primary Afferent Neurons – Sustained and Transient Systems

In summary: the otoliths (and even the semicircular canals) can be thought of as having two co-existing receptor and afferent systems. One is a relatively low frequency system with optimal sensitivity for accelerations but it is relatively insensitive to sinusoidal accelerations above 50 Hz. It is conveyed by neurons with regular resting discharge. These afferents mainly from otolithic receptors in the extrastriolar sensory regions, have a relatively poor response to sound and vibration (Figure 4). The other receptor-afferent system is a transient system (i.e., it is relatively more sensitive to rapid changes in acceleration) conveyed by neurons with typically low irregular resting discharge, conveyed by large diameter fast afferents (Goldberg and Fernandez, 1977; Yagi et al., 1977) and with low threshold and very sensitive responses to sound and vibration) originating from striolar receptors (Figure 4). The important conclusion from physiology is that it is the fast transient system from striola otolithic receptors which is responsible for triggering VEMPs. This sustained/transient division of neural processing in the vestibular system parallels sustained/transient processing in other sensory systems – vision (Cleland et al., 1971; Cleland et al., 1973; Breitmeyer and Ganz, 1976), somatosensory (Hu et al., 2015) and hearing (Andermann et al., 2020).

Ross (1936) originally showed that vestibular afferents were activated by vibration and later (Mikaelian, 1964) and (Young et al., 1977) and others (Wit et al., 1984; McCue and Guinan, 1994; Murofushi et al., 1995) demonstrated that single mammalian and avian vestibular afferents have phase locked activation to sound or vibration, similar to the phase locked activation of primary auditory afferents. Irregular primary otolithic afferents have very short latency to sound and vibration. Figure 5 shows the responses of three single saccular afferents evoked by an air conducted click with a latency to the foot of the action potential of only 0.5 ms – even before the N1 cochlear action potential response to the click (Murofushi et al., 1995) (for reviews see McCue and Guinan, 1997; Curthoys, 2017). However, it must be stated that the latency of the compound action potential (CAP) or wave I of the ABR is influenced by both active and passive components such as the electromotility of the OHCs and mass, stiffness and damping, which adds delays to the auditory system. By contrast, for intense acoustic vestibular stimulation, there is no active filter, and the excitation wave travels relatively quickly and so the latency is short.

Otolithic afferents with irregular resting discharge have poor response to what is regarded as the usual otolithic stimulus – maintained tilts or low-frequency linear accelerations (<50 Hz)—but instead are exquisitely responsive to time rate of change of linear acceleration (jerk) such as occur in vibration. Figure 5 shows how irregular otolithic neurons (blue triangles) have low threshold and steep increase in firing rate to 500 Hz BCV stimuli as the amplitude of the vibration stimulus is increased. In sharp contrast, afferents with regular resting activity either otolithic (orange triangles) or canal (orange circles) simply are not activated even at very high intensities. Regular afferents originate from the extrastriolar region and have sensitive responses to low frequency (<50 Hz) linear accelerations but are not activated by sound or vibration up to high intensities (Curthoys et al., 2006; Curthoys et al., 2014; Figure 5). In summary afferents with regular resting discharge, originating mainly in the extrastriolar region (Goldberg et al., 1990) constitute the low frequency sustained system, whereas afferents with irregular resting activity originating mainly from receptors at the striola constitute the high frequency transient system (Curthoys and Vulovic, 2011; Curthoys et al., 2012) and it is the transient system which is activated by sound and vibration and so is responsible for VEMPs.

### Relating the Neural Responses to the Mechanical Operation of the Otoliths

**FIGURE 6:**
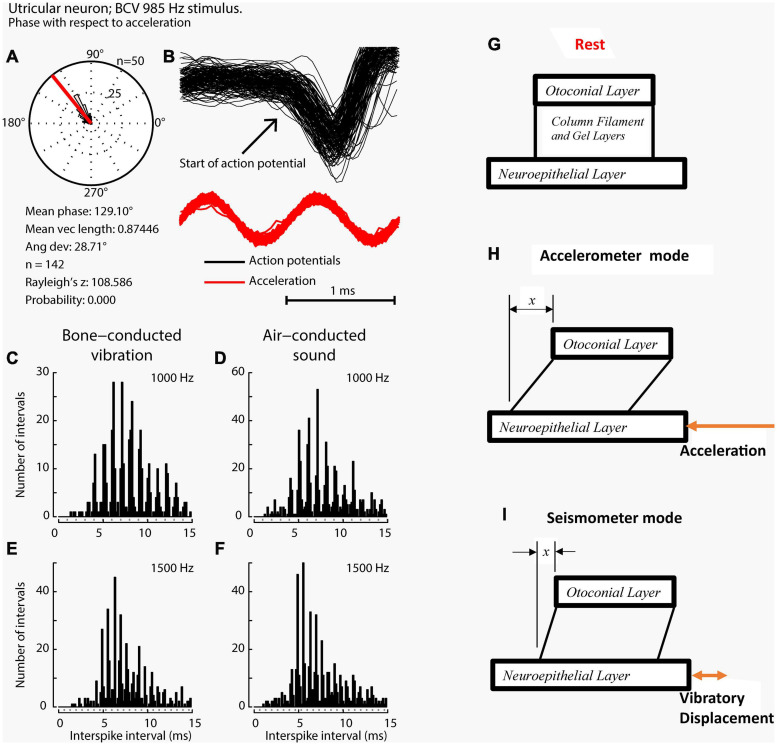
Phase locking of a single primary utricular neuron. **(A,B)** Time series of successive action potentials of the neuron to bone-conducted vibration (BCV) at 985 Hz. Panel **(B)** shows 142 action potentials superimposed and the onset of each successive action potential is shown by the arrow. The red trace shows the *x* channel of the 3D linear accelerometer. The neuron is locked to a narrow phase band of this stimulus. Panel **(A)** shows the circular histogram of the phases of the action potentials clustered around a mean of 129.1°, with angular deviation 28.7°. The test of circular uniformity (Rayleigh’s *z)*, is highly significant showing the probability of a uniform phase distribution is <0.001. The neuron misses some cycles, but when it fires is locked to the stimulus waveform. Reproduced from Curthoys et al. (2019a) with permission of Elsevier. **(C–F)** Histograms of interspike intervals to show phase locking in the same utricular afferent neuron in guinea pig at two high frequencies of BCV **(C,E)** and air- conducted sound (ACS) stimuli **(D,F)**. The bin width is 0.16 ms. The dots below each histogram show integral multiples of the period for the given stimulus frequency. The clustering around these integral multiples demonstrates phase locking at both frequencies. Reproduced from Grant and Curthoys (2017) with permission of Elsevier. **(G–I)** To show the differences in otolithic stimulation between the accelerometer and seismometer modes. **(G)** Show the gross schematic. **(H)** and **(I)** Show the otolithic macula in motion in relation to an inertial reference. In the low frequency accelerometer mode, not only is neuroepithelial layer (NEL) in motion, the otoconial layer (OL) s also in motion lagging behind the NEL due to its inertia, resulting in relative displacement between the two layers. This is also true for the otolith in the seismometer mode **(I)**, where it is the NEL that is in motion with the OL remaining at rest or only slightly in motion, again due to its inertia **(I)**.

The high frequency transient otolithic system can be reconciled with the low frequency sustained otolithic system because it is our contention (Grant and Curthoys, 2017) that otolithic receptors function as accelerometers at low frequencies and as seismometers at high frequencies (Figure 6; Grant and Curthoys, 2017) (This matter is discussed in more detail in section “MECHANICS OF OTOLITHS IN VEMPS TESTING”). At low frequencies, we contend that the neuroepithelial layer (NEL) of the macula is in motion and the otoconia layer (OL) lags behind this movement due to its inertia (the accelerometer mode), whereas at high frequencies the NEL is in a vibratory motion and the OL tends to remain at rest again due to the inertia of the otoconia (the seismometer mode) (Figures 6G,H). Thus the OL has relative motion with respect to the head. At high frequencies in seismometer mode, the NEL has a small vibratory displacement (x), and the OL remains at rest due to its inertia (Figure 6I), again resulting in relative displacement between the NEL and OL and thus receptor hair cell deflection (Grant and Curthoys, 2017). In other words there is a relative displacement between the two otolithic layers at both low and high frequencies. This relative displacement is proportional to the magnitude of the acceleration in accelerometer mode and proportional to the NEL displacement in seismometer mode. For both low and high frequencies, the hair bundles of the receptor are deflected, and the deflection mode is identical at both frequencies (Grant and Curthoys, 2017). The high sensitivity of the type I hair cell bundles in the striolar region pick up the small relative displacements that are imparted in the seismometer mode, whereas the extrastriolar hair cell bundles do not have sufficient sensitivity to pick up these small displacements.

It is unlikely that stimulation causes any deformation of the otoconial layer (OL), but it is possible. Detailed Finite Element Analysis (FEA) models were constructed of the turtle utricular macula and in this analysis there was no indication of deformation of the OL (Davis et al., 2007; Davis and Grant, 2014). These models utilized actual dimensions and shapes of real utricles developed through painstaking anatomical studies from live tissue. These models produce what is called Modal Analysis which shows deformations at various frequencies from motions of the overall OL and sections and parts of OL. This Modal Analysis did not show any deformation of the OL itself below 3 kHz.

### Vestibular Primary Afferent Neurons – Phase Locking of Primary Afferents

When activated by ACS or BCV at frequencies of 250 Hz and above, guinea pig otolithic neurons with irregular resting discharge do not usually generate an action potential on every single cycle (Curthoys et al., 2019a), but the moment when they fire in a cycle is locked to a narrow phase band of the stimulus waveform (Figures 6A–C), in a manner similar to the phase-locking of action potentials in single cochlear afferents to ACS (Rose et al., 1967; Palmer and Russell, 1986; Heil and Peterson, 2017). Phase-locking shows that every single cycle of the stimulus is a physiologically adequate stimulus for primary vestibular afferents, just as it is for cochlear afferents. In any vestibular neuron an action potential may not occur on every single cycle, especially for high-frequency stimuli (>250 Hz), for a number of reasons, such as neuronal refractory period. However the exact moment when the otolithic neuron fires an action potential is tightly locked to a narrow phase band of the imposed stimulus. This is true for both cochlear and vestibular afferent neurons.

For phase locking of the primary vestibular afferent to occur, the hair bundle of the receptor(s) must be deflected and activated once per cycle (at frequencies at or even above 3000 Hz) (Curthoys et al., 2019a). The evidence that this cycle-by-cycle receptor activation does occur is recordings of the cochlear microphonic and more recently the utricular microphonic (UM) – the extracellular hair cell potential caused by otolithic hair bundle deflection (Brown et al., 2017; Pastras et al., 2017a) (see section “ELECTROPHYSIOLOGY”). This important result conclusively shows that high-frequency ‘vestibular’ stimuli (>250 Hz) cause otolithic hair cells to generate potentials in response to these ACS and BCV stimuli (Pastras et al., 2017a; Pastras et al., 2018).

A particularly surprising result is the *precision* of phase locking of primary otolithic neurons (Curthoys et al., 2019a). Irregular vestibular afferents in guinea pigs have a measured phase locking precision to sound and vibration (Curthoys et al., 2019a) which is equal to or even superior to the precision of phase locking of guinea pig cochlear afferents to sound (Palmer and Russell, 1986). Furthermore, this high precision of phase locking of vestibular neurons extends to higher frequencies than reported for cochlear afferents. To appreciate the precision: for a 2000 Hz stimulus, the whole cycle is complete within 0.5 ms, yet the evidence shows that action potentials in some irregular primary otolithic neurons can be locked to a narrow phase band of around 20 deg within that extremely short interval – corresponding to a time window of around 50 microseconds. In short, irregular otolithic afferents have a phase precision in the microsecond time domain. The mechanism for such precision appears to lie in the unique calyx-type I receptor physiology where a very fast process, resistive coupling, may act to confer this very tight phase locking (Contini et al., 2017, 2020).

It is worth noting here that the exact mechanism by which ACS stimulates the otoliths (particularly the saccule), is not entirely clear. In the cochlea, hair cells are displaced due to a pressure gradient across the cochlear partition. In the utricle, it’s possible that ACS induced pressure waves within the vestibule (which is not a fully closed system), also cause pressure gradients across the macula, and thus displacement. For the saccule, which is somewhat anchored to bone, the mechanism is less clear, however we have hypothesized that fluid pressure waves cause a direct displacement of the stereocilia.

When activated by ACS or BCV the action potentials of irregular (transient) afferents show clustering at the integral multiple of the period of the stimulus (Figure 6C). In the example shown, the neuron demonstrated phase-locking up to 1500 Hz for both BCV and ACS. Exactly how hair cell activation occurs is unclear at present. The hair bundles of receptors at the striola project into holes in the otolithic membrane but have tenuous attachment to that membrane. One possibility is that on each cycle the macula movement (i.e., the movement of the NEL) results in endolymph being displaced within the holes in the OL. Because of the dominant viscous flow of the endolymph at these very small dimensions, the hair bundles may track the endolymph displacement almost exactly, resulting in the motion of the hair bundle being directly coupled to the wall motion of the fluid-filled space (the hole) within the OL. Another possibility is that ACS and BCV cause displacement of the macula and that the hair bundles are deflected because they are viscously coupled to the endolymph and OL, and thus there is a differential motion of the hair cell and the hair bundle (Dallos, 1992; Cheatham and Dallos, 1999; Guinan, 2012; Guinan and Nam, 2018; Obrist, 2019; Peterson and Heil, 2020). The implications of these modes of hair cell stimulation are considered in section “MECHANICS OF OTOLITHS IN VEMPS TESTING”.

### A New Clinical Parallel: Auditory Neuropathy and Vestibular Neuropathy

Both the ABR and VEMP depend on synchronous activation of primary afferents and phenomena which interfere with such synchronous activation affect the evoked response. In patients, such an auditory deficit is referred to as auditory neuropathy where, although the cochlear receptors are functioning (and audiograms can appear normal), but the neural response to an abrupt onset stimulus is disturbed as shown by greatly reduced ABR responses to click stimuli (Starr et al., 1996; Michalewski et al., 2005; Kaga, 2016; Moser and Starr, 2016). There is an analogous neuropathy in VEMPs– apparently a vestibular neuropathy—where patients show poor or absent VEMPs (Hu et al., 2020) to repeated clicks or tone bursts. Some of these patients also have auditory neuropathy. The location of the auditory dysfunction was shown by recordings of electrophysiological potentials and the following Section explains the origin of these cochlear and the corresponding vestibular potentials.

The clinical and neural evidence shows that sound and vibration are effective stimuli for (some) vestibular as well as cochlear receptors and neurons. In turn that raises the question of exactly how vestibular receptors are activated by these stimuli and the extent to which vestibular receptor mechanisms have communalities with cochlear receptor mechanisms. The following section explores these similarities and differences in detail.

## Electrophysiology

### Background

**FIGURE 7:**
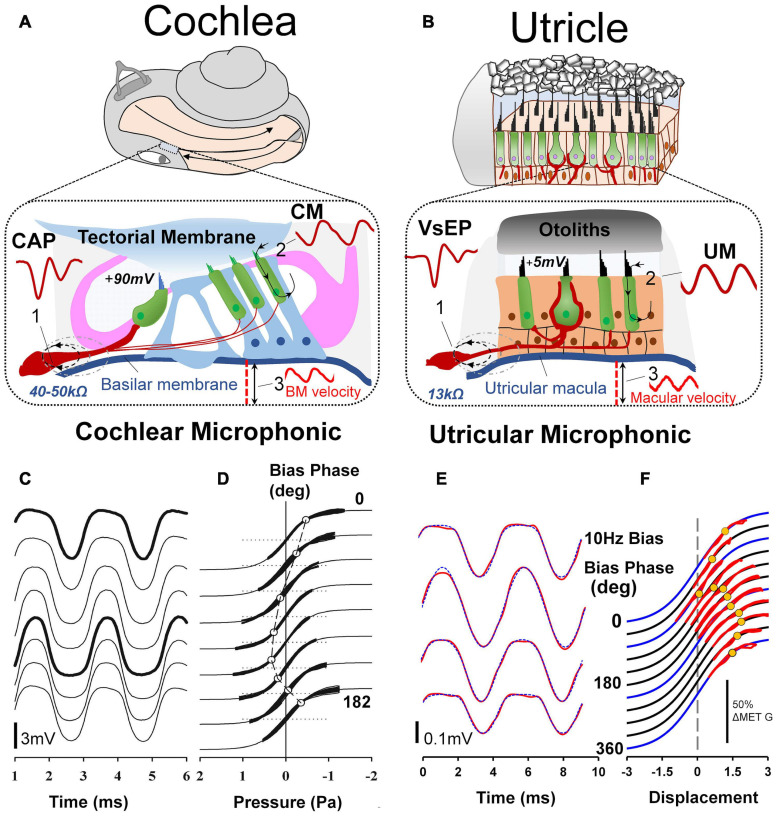
**(A)** For decades auditory researchers have used objective measures of cochlear nerve, hair cell and mechanical function in the CAP, CM, and basilar membrane vibration to comprehensively investigate the cellular basis of hearing and associated disorders. **(B)** Analogous tools have recently been developed and characterized from the utricle, in the VsEP, UM and macular vibration to differentially assess peripheral vestibular function, *in vivo*. Reproduced (adapted) from Assessment of utricular nerve, hair cell and mechanical function, *in vivo*. Pg. 10. Doctoral Thesis. The University of Sydney. Christopher Pastras. 2018. With permission from Copyright owner Christopher Pastras. **(C)** Low-frequency (4.8 Hz) biased cochlear microphonic (CM) waveforms recorded from scala media of the basal turn in response to a 500 Hz, 90 dB SPL probe stimulus after injection of 0.5 μL Healon gel, used to mimic endolymphatic hydrops in the cochlear apex. **(D)** Lissajous figures show the same CM waveforms (heavy lines) plotted against a sinusoidal input, displaced to best fit the transducer curve (thinner line). The open circle indicates the displacement, which corresponds to the operating point at that point on the bias cycle. Reproduced from Salt et al. (2009) Copyright Elsevier (Hearing Research). **(E)** 220Hz Utricular microphonic (UM) waveforms during a 10 Hz hydrodynamic bias of the utricular macula. **(F)** UMs plotted on a Boltzmann Lissajous curve representing MET channel gating, using the approximated macular displacement, which includes an estimate of the operating point with displacement (gray circles). Reproduced from Pastras et al. (2020) Copyright Springer (Journal of the Association for Research in Otolaryngology).

Over the last 90 years inner ear researchers have developed an array of evoked responses to objectively examine cochlear hair cell (HC) and nerve function, and to a lesser extent vestibular HC and nerve function *in vivo*. Although the first of these evoked potentials, the *microphonic*, was recorded around the same period for both the cochlea (1930) (Wever and Bray, 1930) and the vestibular system (1934) (Ashcroft and Hallpike, 1934), the development and uptake of subsequent tools has been vastly different across the two fields. In the auditory field, there has been steady development and use of electrophysiological potentials to investigate cochlear HC and nerve function (Figure 7A). Such potentials include the Cochlear Microphonic (CM), the Summating Potential (SP), the auditory nerve Compound Action Potential (CAP), the Auditory Nerve Neurophonic (ANN) and the Auditory Brainstem Response (ABR). These measures have been used to clarify cochlear operation in experimental animal research, and importantly have progressed to the clinic to diagnose human hearing disorders objectively. Clinical measures include Electrocochleography (ECochG), the ABR, and Otoacoustic Emissions (OAEs).

By comparison, vestibular research has had very few measures available to examine the electrophysiology of peripheral vestibular function, *in vivo*. Primarily, researchers have relied on single neuron recordings, or recordings of the Vestibular short-latency Evoked Potential (VsEP Jones, 1992; Jones and Jones, 1999). Several new experimental measures have recently been recorded from the utricular macula of anaesthetized guinea pigs, including the Utricular Microphonic (UM) (Figure 7B) (Pastras et al., 2017a), Utricular Summating Potential (Pastras et al., 2021), and the Vestibular Nerve Neurophonic. Importantly, these measures provide a first-order, physiological assessment of utricular function related to the clinical indicator, the VEMP. What follows is a direct comparison of *in vivo* evoked HC and neural responses in the cochlea and vestibular system, and how they relate to clinical response measures.

### The Microphonic

In 1930, Wever and Bray documented that sound generated alternating electrical activity from the cat’s cochlear nerve trunk, which closely mirrored the sinusoidal acoustic stimulus (Wever and Bray, 1930). Although initially believed to be action potentials from the cochlear afferents, it was later shown to be due to hair cell activity and so this work was the first recording of the (CM) (Hallpike and Rawdon-Smith, 1934). It only took 4 years to record the analogous potential in the vestibular system (Ashcroft and Hallpike, 1934). Even at this early stage, there were clear differences in response characteristics between measurements in the two sensory systems. Wever and Bray’s ACS CM was sinusoidal between 5 Hz and 12 kHz, whereas, Ashcroft and Hallpike’s non-mammalian ‘VM’, evoked by a tuning fork (and thus a BCV stimulus), was highly complex with double-frequency (*2f*) components and response cancellations, between 50 Hz and 1 kHz (Ashcroft and Hallpike, 1934).

In experimental animals, the CM is routinely recorded via a round window electrode following a simple dorsolateral surgery whereas the VM requires much more complex surgical exposure of the vestibular end organ and ablation of the cochlea to eliminate any cochlea contribution to putative vestibular potentials. This latter point is especially important since in identifying purely vestibular potentials it is mandatory that there can be no possible contribution from cochlear receptors. The majority of VM recordings have been from isolated *in vitro* preparations, which avoid these problems altogether (Corey and Hudspeth, 1979; Corey and Hudspeth, 1983). By comparison, the CM has been recorded in many species, such as the guinea pig (Patuzzi et al., 1989; Patuzzi and Moleirinho, 1998), chinchilla (Drescher and Eldredge, 1974), gerbil (Schmiedt and Zwislocki, 1977), rat (Brown, 1973; Uziel et al., 1981), mouse (Brown, 1973; Cheatham et al., 2004, 2011), and cat (Allen et al., 1971). These mammalian models share similarities with the human cochlea, providing a translational link for understanding human hearing disorders. Results indicate that when recorded at relatively low frequencies, the CM is a passive response primarily generated by the OHCs in the basal turn of the cochlea in close proximity to the recording electrode (Patuzzi et al., 1989).

In 2017, Pastras et al. developed a novel technique to record localized Utricular Microphonics (UMs) from the surface of the utricular macula, of anaesthetized guinea pigs (after cochlea ablation) during BCV and ACS (Pastras et al., 2017a, 2018). This technique is comparable to the CM recording as it provides a localized measure of HC function in the anaesthetized guinea pig, independently of cochlear contribution (Figures 7A,B). There are several important differences between the CM and UM.

Firstly, the hair bundles of the cochlea have uniform polarization, whereas the hair bundles of the utricle have opposite polarities on either side of the striola and face inwards at the striola (Figures 3B,C). For the cochlea, this means that a sinusoidal tone will activate stereocilia in phase and produce receptor currents which sum, resulting in an additive CM response which is large when recorded from ‘near-field’ locations such as the perilymph, and which is also measurable when recorded from ‘far-field’ locations using surface electrodes (Sohmer and Pratt, 1976). For the utricle, a sinusoidal stimulus will activate stereocilia with opposite polarities on either side of the “line of polarity reversal” at the striola, which can result in a complex, semi-cancelled UM. This is especially true when recording from the striola region where the hair bundles ‘switch’ polarity or at ‘far-field’ recording locations, such as the facial nerve canal, where anti-phase UMs sum over a large distance (Brown et al., 2017; Pastras et al., 2017a).

Despite this, a localized UM response can still be recorded in close proximity to the utricular hair bundles at the macula, away from the line of polarity reversal at the striola (Pastras et al., 2017a; Pastras et al., 2020). Like the CM, the ‘near-field’ UM can be used to probe localized changes in otolithic HC function and Mechanoelectrical Transduction (MET) channel gating. Here the localized UM is proportional to the summed current through a local subset of utricular HCs and the extracellular resistance between the current source and the recording electrode. For both cochlear and vestibular HCs, the relationship between MET transducer current and hair bundle displacement follows a Boltzmann activation (sigmoidal) function, and the opening probability of the hair bundle MET channels, or the transducer Operating Point (OP) can be modulated by varying the degree of stereocilia displacement. This has been demonstrated experimentally using a low-frequency biasing technique (Figures 7D,F; Salt et al., 2009; Pastras et al., 2020). Here a slow-dynamic displacement of the basilar membrane or utricular macula, modulates the degree to which cochlear or utricular hair bundles are biased opened (or closed), resulting in changes to cochlear and utricular hair cell sensitivity, and the CM and UM waveshape and saturation (Figures 7C,E).

Although the CM and UM both saturate non-linearly (Figures 7C,E), and a follow a first order Boltzmann function (Patuzzi and Moleirinho, 1998; Pastras et al., 2020), the CM and UM responses have different amplitudes. That is, when recorded from scala tympani, the CM is approximately 1–2 mV, or when recorded from scala media, the CM is several millivolts larger (Figure 7C). By comparison, the amplitude of the UM is about an order of magnitude smaller, and within the 50–500 μV range (Figure 7E). There are several reasons for this difference. Firstly, the differential polarization of the stereocilia in the cochlea or the utricular macula means extracellular receptor currents either sum or partially cancel (Pastras et al., 2017b). The localized UM from the macula is the result of a localized subset of utricular HCs, whereas the CM from the round window measures a much larger population of cochlear HC currents, which sum (in-phase). Secondly, the endolymphatic potential driving the HCs is approximately 20 times larger in the cochlea compared to the utricle (+90 mV vs ∼ 4–5 mV), and surrounding membranes dividing the cochlea scalae have resistances more than twice that of the membranes of the utricular macula (∼45k Ω vs 13k Ω), producing a comparably larger electrical potential for cochlear versus utricular HC stimulation.

Cochlear HCs are also more ‘sensitive’ than vestibular HCs, in the sense they have lower activation thresholds to ACS (and BCV) stimulation. That is, the threshold to obtain a CM response from the round window to sound is approximately 60 dB (i.e., x1000) lower than the sound pressure level needed to obtain a UM to the same stimulus from the utricular macula. Another difference between cochlear and utricular HCs is the difference in their frequency range or bandwidth. Recordings using Laser Doppler Vibrometry (LDV) to measure utricular macula movement in the guinea pig have demonstrated that the UM is most ‘sensitive’ to low-frequency BCV and ACS, between 100 and 200 Hz (Pastras et al., 2018). So, the UM and CM are both sensitive to ‘low-frequency’ stimuli but differ greatly in their bandwidth and dynamic range. The state of the labyrinth during recordings is very different for CM and UM. For the CM, the bony labyrinth generally remains intact, and measurements are taken via non-invasive round window recordings (Brown et al., 2013). This is ideal, as it provides a robust measure of mammalian cochlear HC function with an intact membranous labyrinth, analogous to the physiological state of the labyrinth in the clinic. For the UM, a much more invasive surgical procedure is needed in which the cochlea is ablated via a ventral approach. This eliminates any cochlear contribution to the recorded potential and provides access to the basal surface of the macula in close proximity to the utricular HCs (Pastras et al., 2017a, 2018) and so allows localized UM recording. However it leaves the labyrinth in a dehiscent state, which changes the mechanical operation of the labyrinth (Rosowski et al., 2004) and so modifies the sensitivity of the utricular HCs (Pastras et al., 2018) and afferents (Curthoys, 2017).

### The Summating Potential

The Summating Potential (SP) is used as an objective measure of cochlear HC function in both basic science (Durrant and Dallos, 1974) and clinical research (Gibson, 2009). More specifically, the SP is a stimulus-related DC potential primarily produced by the asymmetry of the gating mechanism on the MET channels of the stereocilia of the HCs, when stimulated by alternating stimuli, which mediates generation of the CM (Choi et al., 2004). Hence, many of the features unique to the CM, are also unique to the SP. For example, the amplitude of the SP is dependent on the size, and specifically the level asymmetry of the CM.

**FIGURE 8:**
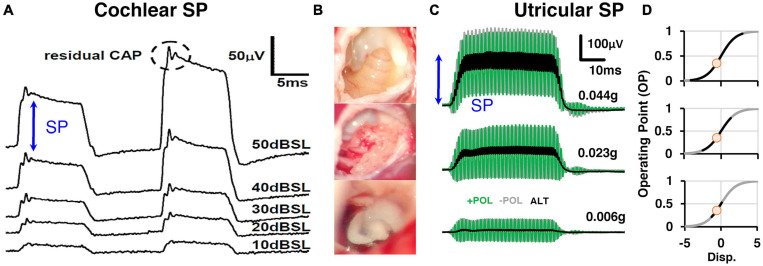
**(A)** Sound-evoked responses recorded simultaneously from scala tympani and scala vestibuli subtracted from one-another to produce differential (DIFF) responses, which is an approximation of the cochlear Summating Potential. DIFF waveforms were evoked by 18 kHz and 20 kHz tone-bursts at a range of sound levels (10–50 dB SPL). Reproduced (adapted) from ‘Origins and use of the stochastic and sound-evoked extracellular activity of the auditory nerve’. Pg. 162. Doctoral Thesis. The University of Western Australia. Daniel Brown. 2006. With permission from Copyright owner Daniel Brown. **(B)** In order to record utricular SPs, a ventral surgical approach is needed to surgically ablate the cochlea and expose the utricular macula for localized recordings on the macular surface. **(C)** Utricular SPs were evoked by alternating BCV stimuli at moderate to intense levels of BCV (0.023 – 0.044 g). **(D)** The generation of SPs were modeled by modulating the MET transducer current into asymmetric (high-slope) regions of the gating profile (first-order Boltzmann function). Reproduced (adapted) from Pastras et al. (2021). Copyright Elsevier (Hearing Research).

Although the SP has been recorded for 70 years in auditory research it has not been previously recorded in the vestibular system. This is likely due to the difficulty in recording pure vestibular HCs responses *in vivo*, independent of any cochlear contribution. We have recently developed a novel method to record localized utricular SPs from the surface of the utricular macula (Pastras et al., 2021; Figures 8B,C). A vestibular SP is expected since vestibular HCs also obey a similar Boltzmann activation function to that of cochlear HCs (Géléoc et al., 1997), where transduction currents saturate nonlinearly for large displacements of the stereocilia bundle (Salt et al., 2009; Pastras et al., 2020). The utricular SP has several common features with the cochlear SP. For example, the generation of the utricular SP also requires moderate to intense levels of ACS and BCV stimulation, where the hair bundles are driven into saturation (Figure 8A vs. Figure 8C). This can be modeled by increasing the stimulation level (and resultant stereocilia displacement) and extent to which the MET transducer current is modulated into asymmetrical (‘high-slope’) regions of the transfer function (Figure 8D). Because the CM and UM input-output curve is not a simple linear function, and is rather sigmoidal, an SP can be produced by large displacements of the HC, which moves the HC I/O function into ‘non-linear’ regions of the S-shaped curve.

Also, like cochlear hair cell responses, the AC:DC ratio of the UM declines with increasing frequency (Russell and Sellick, 1978). This is relevant, because the size of the DC component of the receptor potential (relative to the AC component) dictates the size of the SP. And where there is no DC component, and only an AC component, the response is purely microphonic. Hence, at low-frequencies (e.g., 50–200 Hz), UMs from the macula are relatively more symmetrical compared to higher frequencies (e.g. >300–2000 Hz) Pastras et al., 2021). This result parallels intracellular recordings from the cochlea, in which the AC:DC ratio is largest at low-frequency (100–300 Hz) and declines thereafter with increasing frequencies (Russell and Sellick, 1978). The reduction in AC:DC ratio with increasing frequency is also importantly correlated with the precision of phase locking of primary afferent neurons, where AC receptor potentials are needed for high-precision phase-locking of cochlear afferent neurons or the “Rate code” (Palmer and Russell, 1986). Interestingly (Curthoys et al., 2019a) recently demonstrated that utricular afferents have phase locking at least as precise as cochlear afferents suggesting that the generation of AC receptor potentials are probably relatively similar in both the utricle and cochlea.

The cochlear SP is modulated during mechanical manipulations of the organ of Corti, believed to be largely because to the HC contributions from displacement sensitive OHCs (Pappa et al., 2019). However, recently it was shown the IHCs are also likely embedded into the overlying tectorial membrane (Hakizimana and Fridberger, 2021). This makes the cochlear SP a useful tool to probe mechanical pathologies such as endolymphatic hydrops in MD (using electrocochleography – see below), which shifts the position of the basilar membrane and modulates the sensitivity of these displacement sensitive HCs. Interestingly, the utricular SP is also sensitive to static displacements of the utricular macula (Pastras et al., 2021), meaning that, like the cochlear SP, the utricular SP can also be used to assess mechanical (and morphological) changes in the labyrinth to assess vestibular health and disease.

### Neural Function. The Compound Action Potential

**FIGURE 9:**
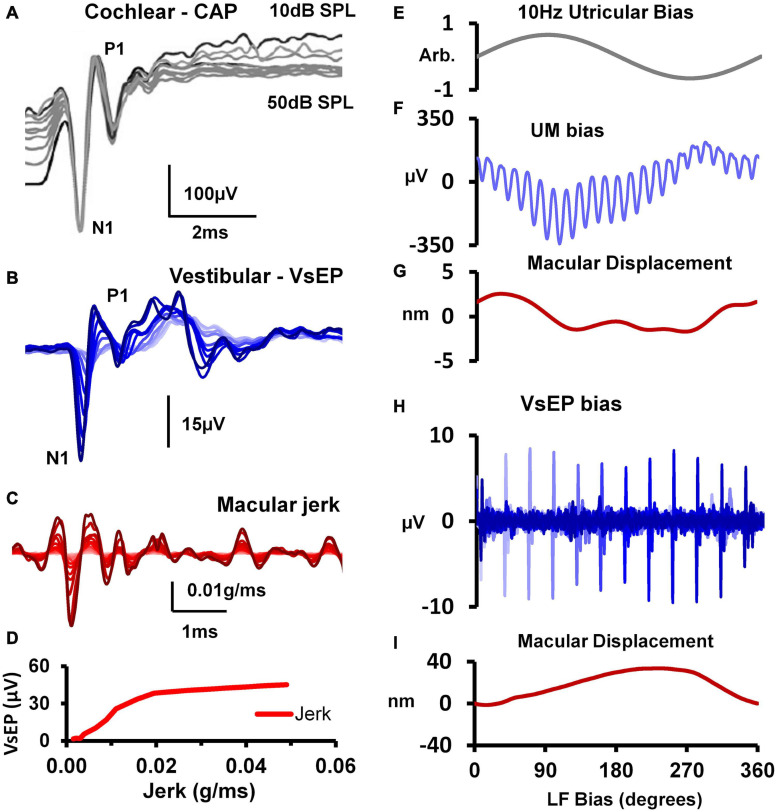
**(A)** Cochlear nerve CAPs evoked by an 18 kHz, 10 ms tone-burst over a 50 dB sound level range above the CAP detection threshold (in 5 dB steps), showing that the amplitude of the N1 potential is unchanged despite such a large change in stimulus intensity. Reproduced (adapted) from ‘Origins and use of the stochastic and sound-evoked extracellular activity of the auditory nerve’. Pg. 153. Doctoral Thesis. The University of Western Australia. Daniel Brown. 2006. With permission from Copyright owner Daniel Brown. **(B)** VsEPs evoked by a 0.6ms BCV pulse (0.3ms rise-fall time) over a 25 dB attenuation range. **(C)** Simultaneous measures of utricular macular jerk (integrated from LDV measures of macular velocity). **(D)** Input-output function for VsEP N1-P1 amplitude against peak-peak macular jerk. Reproduced (adapted) from Pastras et al. (2018) Copyright Elsevier (Hearing Research). 10x more macular displacement is required to modulate the sensitivity of the VsEP compared to the UM. **(E)** 10 Hz hydrodynamic bias of the utricular macula delivered via a fluid-filled pipette sealed into the horizontal semicircular canal (hSCC). **(F)** Cyclic modulation of the UM amplitude evoked by 220 Hz BCV over 1 cycle of the 10 Hz bias (100 ms), and **(G)** the corresponding level of macular displacement. **(H)** Cyclic modulation of the VsEP evoked by a 1ms BCV pulse over 1 cycle of the 10 Hz bias, and **(I)** the associated macular displacement. Adapted from Pastras et al. (2020) Copyright Springer.

The auditory nerve Compound Action Potential (CAP) was first recorded in the 1950s (Tasaki, 1954; Pestalozza and Davis, 1956; Goldstein and Kiang, 1958) and represents the potential caused by synchronous firing of auditory nerve fibres to the onset (or offset) of a stimulus. It has been used extensively in animal models to investigate gross cochlear nerve function and in the clinic in electrocochleography (ECochG) (Eggermont, 1976a,b) and the ABR (Eggermont, 2019) to probe hearing loss. The CAP waveform is comprised of a series of negative and positive peaks, namely the N1, P1, N2, and P2, which resemble a damped 1 kHz sinusoid (Figure 9A) (Brown and Patuzzi, 2010). The cellular origins of these peaks are due to the influx and efflux of sodium and potassium currents through various voltage gated ion channels along the auditory pathway. The longer latency peaks in the ABR response, such as N2, P2, etc., arise from neural activity in the central relays of the auditory pathway such as the cochlear and olivary nuclei, the lateral lemniscus, inferior colliculi (Møller and Jannetta, 1985).

Unlike the low-frequency CM (<500 Hz), which originates ‘locally’ from basal turn hair cells, operating well below their characteristic frequency (CF) and is mostly passive, the CAP has both passive and active elements, depending on stimulus frequency and level. For example, the low-to-moderate suprathreshold, high-frequency (>1 kHz) evoked CAP in the healthy cochlea is generated by HCs operating at the CF and is thus active. This ‘sharp tuning’, boosts cochlear gain by ∼ 60 dB (x1000) and is the result of prestin-mediated somatic electromotility of the OHCs (Cheatham et al., 2004). It is known as the cochlear amplifier, and it effectively negates viscous damping in the cochlea at high frequencies. However, the cochlear amplifier has stable metabolic requirements, which is why the CAP tuning curve loses its sharpness and becomes ‘passive’ following experimental manipulations such as hypoxia and drainage of perilymph (Sellick et al., 1982). This also partly explains hearing loss associated with pathologies such as presbycusis and age-related hearing loss (Ohlemiller, 2002).

The Vestibular short-latency Evoked Potential (VsEP) is the vestibular analogue of the cochlear CAP (Figure 9B) and was arguably first recorded in 1979 by Cazals et al. to ACS in the guinea pig following ototoxic ablation of cochlear HCs (Cazals et al., 1979, 1980; Didier and Cazals, 1989). Following this, the response was recorded in an array of setups and animal models, with early examples including the pigeon (Wit et al., 1981) and the rat (Elidan et al., 1982). The term ‘VsEP’ can be attributed to Josef Elidan and his laboratory, who first described the vestibular compound action potential evoked by abrupt angular acceleration stimuli (∼5000°/s^2^), initially described as the ‘short-latency vestibular evoked response (VsER) (Elidan et al., 1986, 1987a,b) and thereafter as the ‘short latency vestibular evoked potential (VsEP) (Elidan et al., 1991). Since this time the VsEP has been mostly associated with the work of Jones et al. (Jones and Pedersen, 1989; Jones, 1992; Jones et al., 1998; Jones and Jones, 1999) who have recorded VsEPs from the scalp in various animal models using linear up-and-down ‘jerk’ stimulus pulses of short (∼ 2 ms) duration. Other notable techniques have recorded the VsEP to linear acceleration pulses using BCV from the bony facial nerve, producing a more localized field potential (Bohmer, 1995). For a more detailed overview of the VsEP, including its history, stimulation parameters, measurement details (peripheral vs central), interpretation, and techniques to reduce artifacts and cochlear contributions, see Brown et al. (2017).

Like the CAP, the VsEP also represents the synchronous firing of vestibular neurons in response to the onset of the stimulus (Figures 9A,B). And like the CAP, which arises from myelinated primary afferents innervating the IHCs (Brown and Patuzzi, 2010), the VsEP has been shown to arise from irregular primary vestibular afferents, which mostly innervate type I HCs at the striola. This has been inferred through single unit recordings, in which it is the irregular calyx/dimorphic afferents which respond to transient stimuli such as BCV and ACS (see section “INTRODUCTION” above) (Curthoys et al., 2016), unlike regular bouton afferents, which respond to ‘low-frequency’ sustained stimuli, but not to sounds or vibration. More specifically, the VsEP has been shown to be sensitive to kinematic jerk (Jones et al., 2011)of the animal’s head frame (Figures 9C,D). Kinematic jerk is the time rate of change of linear acceleration. It is still not clear how the HC generators of the VsEP are activated during stimulation. In the cochlea, there are several distinct micromechanical activation modes of receptor HCs, where displacement sensitive OHCs are activated by reticular lamina-tectorial membrane shearing, and the IHCs are stimulated by longitudinal and radial flow, which also become entrained with partition displacement at higher frequencies via viscous drag. However, recently it has been revealed that IHCs are likely embedded in the tectorial membrane, and may be displacement sensitive like OHCs (Hakizimana and Fridberger, 2021). Ultimately, the CAP is produced by both HC subtypes synergistically working together, where displacement sensitive OHCs amplify basilar membrane vibration and increase the input drive to the IHCs, which directly branch to myelinated spiral ganglion afferents, which generate the CAP response.

In terms of the micromechanical activation of vestibular HCs responsible for generating the VsEP, it is possible that their stereocilia are viscously coupled. That is, recent immunohistochemistry studies in the mouse utricle have shown that striolar hair bundles are short and stiff, and not physically attached to the OL, whereas the extrastriolar bundles are longer and appear to be embedded in the membrane (Li et al., 2008). This suggests that HCs responsible for generating the VsEP (i.e., striolar, type I HCs) may be viscously activated, similar to IHCs in the cochlea. Furthermore, a recent study which modulated the macromechanics of the macula using low-frequency hydrodynamic biasing (<20 Hz), required 10 times less macular displacement to modulate the sensitivity of the UM, compared to the VsEP response (Figures 9E–I; Pastras et al., 2020). This suggests that the UM is more likely displacement sensitive, whereas the VsEP is likely velocity sensitive. This aligns with previous work, where the UM scales with macular displacement across frequency (Pastras et al., 2018; Pastras, 2018), and not velocity - the saturation of the UM (and amplitude of the utricular SP) are modulated following static displacements of the utricular macula (Pastras et al., 2021). Taken together, these results demonstrate that the UM and VsEP can potentially be used to differentially assess distinct cellular substrates of utricular pathology, similar to how the CM and CAP are used to assess different regions of the cochlea frequency tuning curve (e.g., tail vs CF).

There is no conflict between velocity sensitivity at the micromechanical level and the jerk sensitivity of the VsEP that Jones et al. showed (Jones et al., 2011). They reported that the effective stimulus at the level of the skull is jerk, but due to the micromechanical operation at the level of the utricular macula, that jerk stimulus at the skull results in velocity of the stereocilia probably being the effective stimulus for the receptor hair cell.

Recently, dual patch recordings of the type I hair cell and calyx afferent have implicated 3 distinct modes of synaptic transmission at this unique synapse: glutamatergic quantal transmission, K+ accumulation, and resistive coupling (in the microsecond time domain). These 3 modes of synaptic transmission may explain some features of the VsEP, such asrapid response kinetics, relative insensitivity to forward-masking, unlike the cochlear CAP (i.e., the VsEP can be recorded at high stimulation rates such as 125 Hz or 8 ms between pulses, whereas the CAP is largely attenuated at these rates). Hence, there may be no need for a somatic-electromotility in the vestibular system, which in the viscous environment of the cochlea is suited to counteracting damping, especially at ‘high-frequencies’. At the very least, what is needed in the otoliths are highly sensitive linear accelerometers, which operate over a physiological relevant bandwidth, from gravity to several kilohertz (see section “MECHANICS OF OTOLITHS IN VEMPS TESTING”). Displacement and velocity sensitive vestibular HCs, coupled with multiple synaptic modes of transmission, with top-down feedback from the efferents can in theory, provide this.

The VsEP can be recorded via an electrode in the facial nerve canal, near the superior branch of the vestibular nerve. This technique provides a greater electrical pick up from the primary afferent neurons and a relatively large evoked potential (∼30–50 μV). However, this response is evoked using BCV and ACS, which also stimulates the cochlea. Hence, in order to record VsEPs uncontaminated by cochlear potentials with the labyrinth intact, the cochlea must be silenced whilst also being kept structurally intact. Masking noise has been used previously to minimize cochlear contribution, and although this does a good job at disrupting the synchronized afferent activity from cochlear neurons, it does not fully eliminate receptor potentials such as the CM and SP (Deatherage et al., 1957; Marsh et al., 1972). Hence, a more reliable method is to chemically silence the cochlea using slow perfusion of KCl, whilst sparing vestibular function, as recently demonstrated in Pastras et al. (2020). Using this technique, VsEPs can be recorded from the facial nerve canal with the cochlea structurally intact, yet functionally inactive.

### Electrocochleography vs. Electrovestibulography

When a combination of HC and neural field potentials are recorded together in response to ACS or BCV bursts, the technique is called Electrocochleography (ECochG) in the cochlea, or Electrovestibulography (EVestG) in the vestibular system. For the reasons listed above, ECochG is relatively straightforward to record in experimental animal models at locations such as the round window, where there is a large electrical pickup from both cochlear HCs and neurons. Moreover, ECochG has also been used for several decades in the clinic to differentially assess HC vs nerve dysfunction associated with hearing loss. However, more reliable and robust measures such as the ABR and Otoacoustic Emissions (OAEs) are taking its place.

**FIGURE 10:**
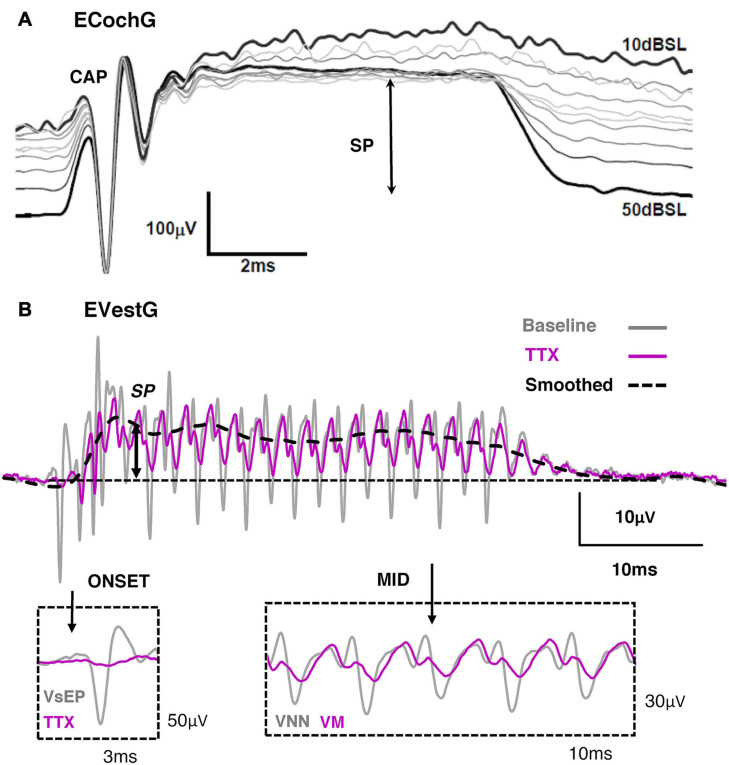
**(A)** Electrocochleography (ECochG) response recorded from the round window of the guinea pig during an 18 kHz, 10ms tone burst of varying intensity. Reproduced (adapted) from ‘Origins and use of the stochastic and sound-evoked extracellular activity of the auditory nerve’. Pg. 153. Doctoral Thesis. The University of Western Australia. Daniel Brown. 2006. With permission from Copyright owner Daniel Brown. **(B)** Electrovestibulography (EVestG) response taken from the facial nerve canal of the guinea pig to 507 Hz, 40 ms BCV train burst. Tetrodotoxin (TTX) abolished the onset VsEP (gray) and the mid response VNN (gray), leaving behind the VM and SP. Reproduced (adapted) from Pastras et al. (2021) Copyright Elsevier (Hearing Research).

Depending on the stimulus, ECochG will contain varying levels of an onset CAP, an Auditory Nerve Neurophonic (ANN), SP and CM. For example, if the acoustic stimulus is well beyond the HC corner frequency, such as in Figure 10A with an 18 kHz tone, the CM will be largely filtered, leaving behind the SP and CAP. Moreover, if the tone exceeds the frequency for cochlear nerve synchronicity (i.e., beyond 1–2 kHz), the ANN will be non-existent. That is the reason why in Figure 10A only the onset CAP and SP can be detected at 18 kHz. Likewise, if the acoustic stimulus is alternated, the fundamental frequency of the CM will be largely cancelled, leaving behind the CAP, ANN, SP and any higher-order harmonic components. Additionally, the presence of the onset CAP is dependent on the rise-time of the tone burst, where a long rise-time (and highly smoothed stimulus envelope) can smear the synchronous activation of afferents and greatly diminish the onset (or offset) CAP. This helps to explain the clinical observation that VEMPs are largest to stimuli with very short rise times (Burgess et al., 2013), and that VEMPs can be activated by a multitude of transient stimuli such as tone-bursts, chirps, clicks, taps and pulses. The EVestG has been shown to contain a combination neural and HC components, which include the onset VsEP, Vestibular Nerve Neurophonic, Vestibular Microphonic and Vestibular Summating Potential.

### Peripheral Electrophysiology in Experimental Animal Models and Their Link to Clinical Recordings

Clinical vestibular researchers have used an ‘indirect’ measure of peripheral otolith function in the VEMP (see section “INTRODUCTION”). The VEMP can be thought of as the clinical myogenic correlate of the VsEP, which is, similarly, driven by irregular primary afferents at the striola (Curthoys et al., 2019b). Hence, many of the features which apply to the VsEP (such as transient response activation), should also apply to sensory receptors generating the VEMP at the striola.

Moreover, it is possible to understand how otolithic HCs operate in the VEMP recording, from measurements of the otolithic microphonic during BCV and ACS. That is, by looking at the sensitivity and frequency range of otolithic microphonics from the macula, one can begin to understand the cellular correlates of the VEMP. Results indicate that otolithic HCs are active up to 5 kHz BCV and ACS (Pastras et al., 2018). Hence, it is no surprise that ‘high-frequency’ VEMPs can be recorded for stimulus frequencies beyond a kilohertz, and very clearly in patients after an SCD (Manzari et al., 2013; Curthoys and Manzari, 2020).

Recent results using simultaneous LDV recordings of macular velocity and the UM indicate that the utricular HCs have different macromechanical activation modes for BCV and ACS (Pastras et al., 2018). Hence, it is possible that the sensory activation of receptors during the VEMP is different for BCV compared to ACS (Govender et al., 2016) and some clinical evidence supports this difference (Govender et al., 2016). This may have clinical implications for the future diagnosis of vestibular disorders, such as endolymphatic hydrops in MD, where the tuning of VEMP shifts upwards to 1 kHz (see also sections “INTRODUCTION” and “MECHANICS OF OTOLITHS IN VEMPS TESTING”). For example, if the macromechanical activation pathways of the VEMP are different for sound and vibration, then the change in tuning may also be different for both stimulation modes, depending on pathology and the mechanical (or morphological) change.

**BOX 1. Undamped Natural Frequency – Non-Mathematical Description.**

The undamped natural frequency (UDNF) of otoliths defines their dynamic behavior when it is excited by a high frequency stimulus in VEMPs testing (Curthoys and Manzari, 2020; Manzari et al., 2013; Noij and Rauch, 2020). Note, that “high frequency” with regards to vestibular stimulation (and the VEMP) is >500 Hz, which is much lower than what is considered high frequency in the cochlea.

The UDNF is defined as the square root of shear stiffness of the gel-column filament layer, divided by the mass of the otoconial layer (OL). When these two quantities, shear stiffness and OL mass, are numerically evaluated they are both functions of the utricle surface area (area defined by its perimeter), and because the stiffness is divided by the mass, the area divides out. The resulting expression for the UDNF is then the square root of the shear stiffness defined by the shear modulus of the gel-column filament layer which is divided by the product of the OL density, the thickness of this layer, and the thickness of the gel-column filament layer.

The thickness of the two layers, OL and gel-column filament layer, primarily define the UDNF of the system. The other two variables, gel-column filament layer shear modulus and OL density, are less variable and appear to remain somewhat constant. The result is that for a healthy young adult the UDNF is predicted to be 400 Hz. This frequency is dependent on the parameter values under the square root, and these parameters are difficult to measure experimentally.

The excitation frequencies for VEMP testing results in the utricle behavior as a seismometer, as opposed to an accelerometer at lower excitation frequencies (Grant and Curthoys, 2017). In seismometer mode, the OL essentially remains at rest due to its inertia, while the neural-epithelial layer is in motion, shearing the gel-column filament layer. This relative shear displacement is proportional to the head displacement imposed by the high frequency VEMP stimulus. It is this shear displacement that deflects hair cell bundles, either by their attachment to the OL and/or the bundles being dragged and forced through their surrounding endolymph, opening ion-channels and initiating neural signals.

Using the UDNF and the fact that the utricle system is underdamped (Dunlap and Grant, 2014) a curve predicting the relative shear displacement between the two layers, otoconial and neural-epithelial, relative to a unit neural-epithelial layer displacement, can be constructed as a function of stimulus frequency. This curve is called a Frequency Response Curve or Transfer Function Plot and is shown in Figure 12. This curve remains flat at zero displacement until the excitation frequency near the UDNF. The curve then rises rapidly as the excitation frequency increases and passes through the UDNF, continues to rise, and then flattens out with increasing excitation frequency. Because the system is underdamped, the curve has a slight upward trend or upward bulge, before it begins to flatten out beyond the UDNF. The highest point on this part of the response curve is defined as the best test frequency or just best frequency. This exact point in the response curve would be difficult to detect in VEMP testing.

In summary, basic physiological measures from the utricular macula, can be used to differentially assess utricular nerve and hair cell function, and additionally, be utilised to investigate the response characteristics of the VEMP as a means to understand vestibular health and disease.

## Mechanics of Otoliths in VEMPs Testing

### Undamped Natural Frequencies

The following shows how knowledge of the characteristics of the layers of the utricular macula (thickness, stiffness etc.) can be used to predict the response of receptors and afferents to stimuli of varying frequency [For a non-mathematical description of Undamped Natural Frequency (UDNF), see Box 1 text]. The layers of the macula have been simplified to the otoconial layer (OL) and neuroepithelial layer (NEL) and between them is the shear layer comprised of the Gel layer and the column filament layer (CFL) (see Figures 6G–I for a simplified schematic).

The UDNF ω_*n*_ can be predicted from the Shear Layer (SL) stiffness *k*_*SL*_, and the Otoconial Layer (OL) mass *m*_*OL*_, using the standard expression for UDNF

(1) $$\omega_{n}=\sqrt{\frac{k_{SL}}{m_{OL}}}$$

Note that this is a shear oscillatory frequency which results in the OL moving parallel to the Neural-Epithelial Layer (NEL).

The shear layer includes the Gel Layer (GL) which lies next to the OL, the Column Filament Layer (CFL) which rests on the NEL, and Hair Cell Bundles (HCB) which reside in both layers. The shear layer stiffness used here is an effective stiffness that includes both the GL, CFL, and the HCB stiffness collectively, and includes the entire thickness of both layers. The GL is relatively thin compared to CFL and most of the effective stiffness is contributed by the CFL and HCB. The stiffness of this effective shear layer is expressed as

(2) $$k_{SL}=\frac{F}{\delta }$$

where: *F* = a shear force acting on the surface between the SL and OL, and δ = the relative displacement between the NEL and OL produced by the force *F*. The force *F* is hypothetical here, in an actual stimulus it is the NEL that is moving due to head motion, and the OL remains at rest due to its inertia. This force *F* is the resultant of a shear stress τ acting over the entire surface between the OL and SL, designating this area *A*, which defines the force in terms of shear stress and area *F* = τ*A*. The shear stress can be defined in terms of the shear modulus *G* which is defined as

(3) $$G=\frac{\tau }{\gamma }$$

where: g = the shear strain, which is δ = deflection divided by shear layer thickness *t*_*SL*_, $\gamma =\frac{\tau }{t_{SL}}$

Combining these expressions, the SL stiffness expression becomes

(4) $$k_{SL}=\frac{F}{\delta }=\frac{G\gamma A}{\gamma t_{SL}}=\frac{GA}{t_{SL}}$$

The shear modulus *G* represents a material property of the effective shear layer and can be related to Young’s modulus.

The OL mass *m*_*OL*_is the product of the density of the OL, ρ_*OL*_ and the volume of the OL, *V*_*OL*_ = *t*_*OL*_*A*

where: *t*_*OL*_ = the thickness of the OL, and the mass then is

(5) $$m_{OL}=\rho_{OL}At_{OL}$$

The UDNF becomes

(6) $$\omega_{n}=\sqrt{\frac{k_{SL}}{m_{OL}}}=\sqrt{\frac{\frac{GA}{t_{SL}}}{\rho_{OL}At_{OL}}}=\sqrt{\frac{G}{\rho_{OL}t_{OL}t_{SL}}}$$

and the area divides out. The utricle and saccule area are probably larger to incorporate more HC and thus more neural input for the vestibular system. With numerical values for the parameter under the square root sign a prediction of the UDNF of an otolith can be made.

Numerical parameter values for predicting the human UDNF, those under the square root sign of Eqn. 6, will be evaluated. Most of the parameter values in Eqn. 6 have been evaluated in animals and in some humans. Thickness of the shear layer ***t****_*SL*_* has not been studied in any animal or human.

### Shear Modulus - G

The shear modulus has never been measured directly in any animal, including humans, but evaluations have been made using indirect experimental measures. The best experimental measurement was done in the turtle (Dunlap and Grant, 2014) where the shear modulus was 9.42 Pa (with 95% confidence interval of 8.36 – 10.49) in the medial-lateral direction, and 11.31 Pa (10.21 – 12.41) in the anterior-posterior direction. An overall mean value of 10 Pa (9.3 – 11.5) will be used here. Other researchers are in approximate agreement with these values. Shear Modulus values measured by others, converted from Young’s modulus using an incompressible material, are all for the canal cupula except one and they are: *G* = 7 Pa in zebra fish (McHenry and van Netten, 2007); *G* = 11.53 Pa in pike fish (ten Kate, 1969), a human cupula model estimates of *G* = 3.3 Pa (Selva et al., 2009), and a human otolith model estimate of the order *G* = 3.3 Pa (Kondrachuk, 2001).

### Otoconial Layer Density - ρ_*OL*_

The OL density is based on a 4:1 calcite crystals (ρ = 2710 kg/m^3^) to gel ratio (ρ = 1003 kg/m) for this layer (Pote et al., 1993). The otoconial crystals in the OL are in the calcite crystal form, and these crystals are bound together by the same protein gel material from the GL. When the five parts (4 parts crystals and 1 part gel) of the OL are considered, the density is ρ_*OL*_ = 2368 kg/m^3^

### Otoconial Layer Thickness - t_*OL*_

**FIGURE 11:**
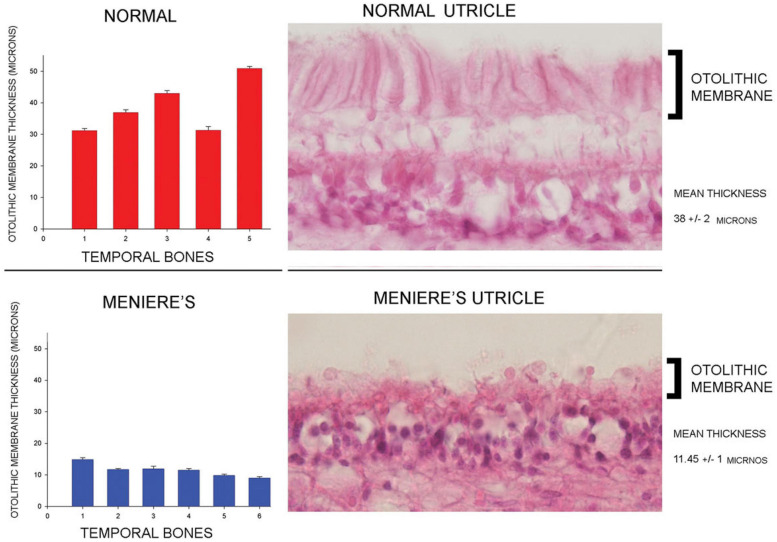
Thickness of the human Otoconial Layer. The Normal Utricle photomicrograph shows otoconial layer thickness values from five specimens; The lower Menière’s utricle showing diseased macula with a much thinner otoconial layer, photomicrograph and OL thickness for six specimens. Reproduced by permission from Ishiyama et al. (2015).

Value for human thicknesses that will be utilized here is: *t*_*OL*_ = 38 ±2 μm (Ishiyama et al., 2015), which is the most accurate available. This thickness is shown in the Figure 11 photomicrograph along with the five temporal bone values used for the mean.

**FIGURE 12:**
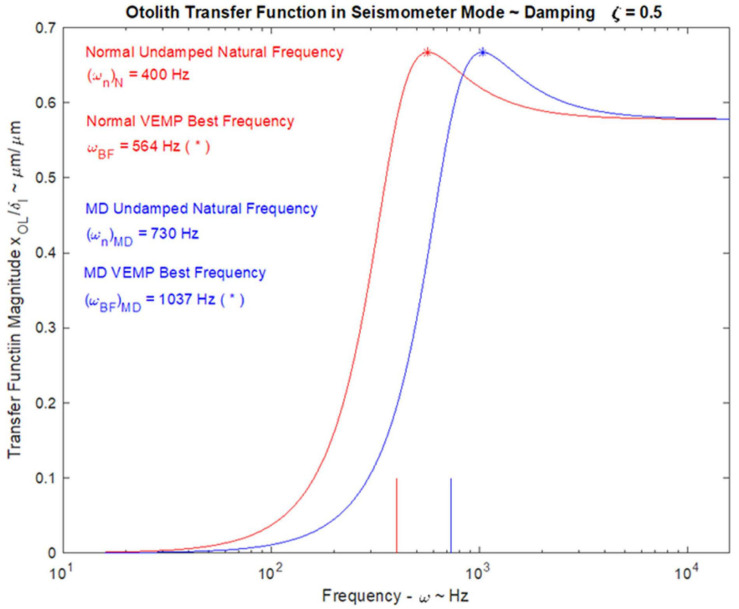
Utricle frequency response (Bode Plot) in Seismometer Mode (shown in red) using estimated UDNF of 400 Hz (shown as vertical red line) and damping ratio of ζ = 0.5 (Dunlap and Grant, 2014). The best test frequency is 564 Hz, shown as red asterisk (*). The frequency response curve for Menière’s Disease (shown in blue) using and estimated UDNF of 730 Hz and with a best frequency of 1037 Hz shown as blue asterisk (*).

### Shear Layer Thickness - t_*SL*_

Human shear layer thickness has not been measured or reported in any published research. The value has been evaluated from two photomicrographs of human specimen obtained from Ulf Rosenhall (1972). The mean of multiple scaled values from the photomicrographs with a 10% allowance for tissue processing shrinkage was ***t***_*SL*_ = 17.5 μm. The shrinkage allowance may not be sufficient for this type of gel tissue; however, it is the standard value currently in use. Also scaling from the photomicrograph histological section shown in Figure 11 with a 10% shrinkage allowance was a tenth of a micron larger. The value of ***t***_*SL*_ = 17.5 μm will be used here.

Using the above mean values, the UDNF is

(7) $$\omega_{n}=401Hz$$

Using the 95% confidence interval values for *G* and the two values on either side of the mean for OL thickness results in the following spread in the value above using the means

(8) $$\omega_{n}=377Hzand441Hz$$

The parameter values utilized are the best that can be found from current literature. A value for the human UDNF of ω_*n*_ = 400 Hz will be used here for further analysis.

### Otolith Seismic Mode Transfer Function and Frequency Response

A transfer function of Neural Epithelial Layer (NEL) motion, with the utricle in seismometer mode induced by a Bone Conducted Vibration (BCV) stimulus will show the best frequency for maximizing output for clinical testing. Using the mean value for UDNF of ω_*n*_ = 400 Hz and a damping ratio of ζ = 0.5 (Dunlap and Grant, 2014) for an underdamped system, both estimates for humans, will be used.

The transfer function is developed from the basic linear differential equation that describes the relative displacement motion *x* between the NEL and the OL. That basic equation is

(9) $$m\ddot{x}+c\dot{x}+kx=\left(1-\frac{\rho_{e}}{\rho_{OL}}\right)mA_{GI}$$

where: *x* = relative displacement between OL and NEL, the over dots represent differentiation with time, *m* = OL mass, *c* = SL damping coefficient, *k* = SL stiffness coefficient, ρ_*e*_ = endolymph density, ρ_*OL*_ = OL density, B = $\left(1-\frac{\rho_{e}}{\rho_{OL}}\right)$ = buoyancy term, and *A*_*GI*_ = gravitoinertial acceleration (sum of NEL acceleration and gravity) (Grant and Curthoys, 2017). Dividing by the OL mass the basic equation is reduced to two parameters and a buoyancy term

(10) $$\ddot{x}+\frac{c}{m}\dot{x}+\frac{k}{m}x=BA_{GI}$$

This governing equation of motion for the OL relative to the NEL is converted using nomenclature for standard dynamic system parameters: Damping ratio $\zeta =\frac{c}{c_{o}c}$, where: *c*_*o*_ = critical damping, and *z* < 1 is an underdamped system, $\omega_{n}=\sqrt{\frac{k}{m}}$ (the UDNF as introduced above), and Eqn. 10 becomes

(11) $$\ddot{x}+\left(2\zeta \omega_{n}\right)\dot{x}+\left(\omega_{n}^{2}\right)x=BA_{GI}$$

The above is converted into a transfer function for an utricle operating in seismometer mode:

(12) $$\frac{x}{D_{NEL}}=B\left(\frac{\omega^{2}}{\left(\omega_{n}^{2}-\omega^{2}\right)+j\left(2\zeta \omega_{n}\right)}\right)$$

where: *D*_*NEL*_ = displacement of the NEL relative to an inertial frame of reference, *x* = relative displacement between NEL and OL, $j=\sqrt{-1}$ = imaginary number, and ω = the excitation or stimulus frequency [for details of this derivation see Grant and Curthoys (2017)].

Constructing a frequency response diagram using Eqn. 12 for the otolith in seismometer mode, will have the utricle measuring the displacement of the NEL’s motion. The frequency response diagram is shown in Figure 12 (red curve) for an utricle operating above its UDNF. The Figure 12 plot, with gain $\frac{x}{D_{NEL}}$ vs stimulus frequency ω, was done using an UNDF ω_*n*_ = 400 Hz (human value estimated above), and a damping ratio ζ = 0.5 [value measured in the turtle (Dunlap and Grant, 2014)]. The seismometer transfer functions peaks at its best frequency for use in VEMPs testing. This best test frequency is defined as the one which produces the greatest gain on the transfer function curve. This greatest gain displacement would give hair cell bundles their greatest displacement for a given stimulus displacement magnitude, producing the best neural stimulus. The best test frequency is ω_*BF*_ = 564 Hz, as seen in Figure 12, and this value is very near the most commonly used in VEMPs testing frequency of 500 Hz. Also seen in the figure is that the range of best frequency is broad from 500 to 600 Hz. There is such a modest increase in gain over this stimulus frequency range, that it would be improbable to detect the best test frequency with an VEMP test.

### Menière’s Transfer Function - Otolith in Seismic Mode

Recent published research shows that aging patients with Menière’s Disease (MD) have decreased OL thickness and thus decreased mass, resulting in higher UDNFs (Ishiyama et al., 2015). This deficiency has been identified using higher stimulus frequencies for VEMPs to produce larger responses in aging subjects (Rosengren et al., 2019). These results are explained by the decreased OL thickness shown in postmortem evaluation of patients with Menière’s Disease (Ishiyama et al., 2015). Importantly, this thickness change is likely to be related to the chronic/sustained dysfunction of hearing and balance in MD sufferers, and is unlikely to be involved in the fluctuation of symptoms (e.g., hearing fluctuation).

Using the MD decrease in OL thickness from 30 down to 11.45 μm shown in Figure 11, and leaving all other parameters unchanged, the UDNF increased from 401 to 730 Hz. Using this MD UDNF for a frequency response plot moves the whole curve to the right resulting in a higher frequency response as seen by the blue curve in Figure 12. This moves the best frequency point for normal individuals of 564 up to 1037 Hz for MD patients. From this exercise it is clearly seen that it is this change in OL thickness, decreasing the OL mass, that is probably causing the larger VEMP response at increased stimulus frequency. This loss of mass in the OL is a change in the peripheral mechanics, and this change is seen in the neural response to a VEMPs stimulus. A fundamental principle involved here, and for any sensory system (biological, mechanical, electronic, etc.), the system cannot obtain any information that is not contained in the primary signal of the system sensor. In this case the system sensor is the otolith which has two modes: accelerometer and seismometer. In the case of MD examined here, a fundamental change in the system UDNF changes the whole system frequency response. It remains to be seen if the upward shift of VEMP tuning in MD patients is related to their low frequency hearing loss.

### Transient Behavior of Otoliths in VEMPs Testing

It should be recognized that Frequency Response Diagrams as addressed in the previous section represent the steady state response to a stimulus, and any transient response to a stimulus is not reflected in these diagrams. This steady state response is by definition and by design in frequency response diagrams. Transient behavior is not represented or depicted in any way in these diagrams. The utility and mathematics for Frequency Response Diagrams or Bode Diagrams (sometimes called Bode Plots) were worked out back in the 1930s by WH Bode at Bell Labs. These diagrams were intended for use in electronic circuits and transmission line losses, as well as in control systems design and behavior work, where transient behavior was not an issue. The transient response in VEMPs testing is significant and is addressed in this section.

**FIGURE 13:**
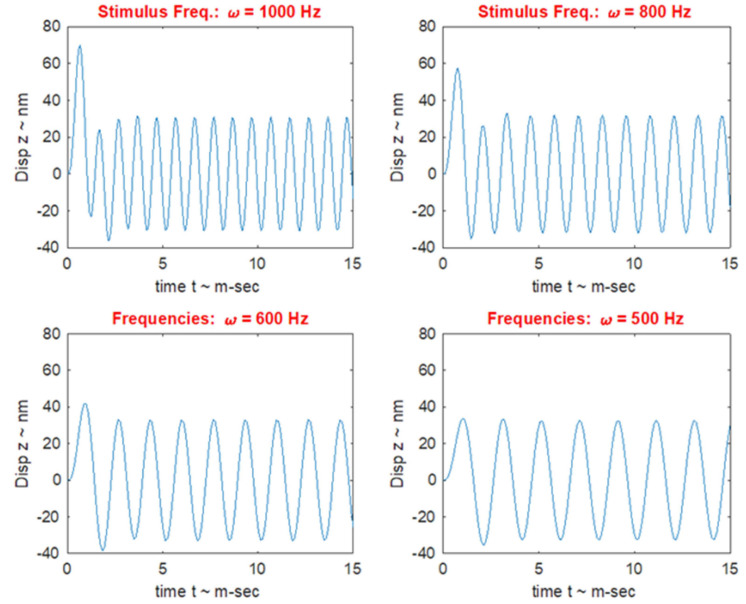
Relative Displacement *z* vs. time *t* response curves, for solutions of Eqn. 11, the equations of motion for relative displacement between OL and NEL. Parameter for this solution: UDNF of 400 Hz (human estimate from above), damping ratio of 0.5 (value measured in the turtle; Dunlap and Grant, 2014), and with a NEL maximum displacement of 50 nm (estimate for a VEMP stimulus; Grant and Curthoys, 2017). The solutions were obtained numerically using MATLAB and show significant transient displacement response at the onset of the response when the stimulus frequency ω, indicated in red above each curve, is well above the UDNF. As can be seen in the 1000 and 800 Hz curves, the transient displacement is above the maximum stimulus value of 50 nm. The transient is present with a 600 Hz stimulus, and almost absent at 500 Hz.

Solving Eqn. 11, which describes the dynamic behavior of relative displacement between NEL and OL of otoliths in its seismic mode, was integrated numerically in time using MATLAB for these solutions. The NEL stimulus used in this simulation was a sinusoidal displacement of NEL, with maximum displacement of 50 nm, and with various stimulus frequencies ω. The response shows a significant displacement at initiation of NEL motion stimulus with stimulus frequencies well above the UDNF. These results are shown in Figure 13, using the following parameters: UDNF ω_*n*_ = 400 Hz (human value estimated above), and a damping ratio ζ = 0.5 (value measured in the turtle Dunlap and Grant, 2014).

These results indicate that it is more advantageous to use 1000 Hz stimulus with zero rise time rather than the 500 Hz with a 2ms rise time that is in customary use for VEMPs testing. A significant transient displacement occurs in less than 5 ms, with amplitude that is 1.4 times the NEL displacement stimulus. This transient stimulus has settled down to a steady state displacement after 0.5 ms time. Most VEMPs testing today incorporates a ramping up to the maximum displacement used for the stimulus, and these ramp times are generally in the 2–5 ms range. It is also seen that this ramp up to maximum stimulus magnitude should also be avoided to capture this transient behavior. By doubling the customary frequency of 500 Hz and eliminating the ramp up to maximum stimulus frequency, significant deflection stimulus to striolar hair cell bundles could be achieved. The significant contribution of rise time to oVEMP amplitude is shown in Figure 1C.

### Threshold Displacement

**FIGURE 14:**
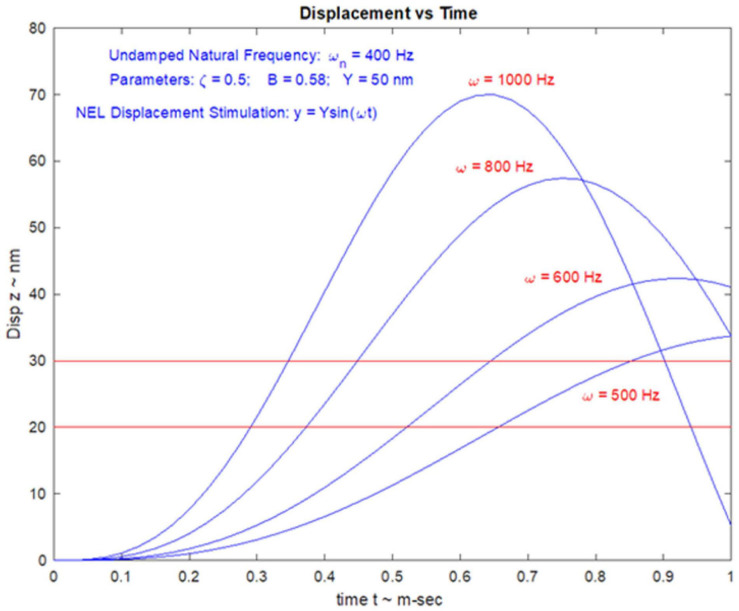
Relative displacement *z* vs. time *t*, over 1 ms, for various stimulus frequencies. Suspected threshold range of 20–30 nm is shown in red (Grant and Curthoys, 2017). The two higher frequencies reach the threshold range before 0.5 ms, while the lower two reach this range in in a time frame greater than 0.5 ms. This figure shows a better illustration of the maximum or peak values for the transient behavior.

Stimulus frequency also influences the time before relative displacement has reached the threshold displacement of hair cell bundles. Using the same simulation for stimulus frequency in the previous section (ω_*n*_ = 400 Hz, ζ = 0.5, and *D*_*NEL*_ = 50 nm) and examining the transient response over a shorter time period, shows that for higher frequencies there is shorter time to reach the range of suspected threshold deflection for striolar hair cell bundles. This is shown in Figure 14.

The suspected threshold for bundle displacement is expected to be in the 20 – 30 nm range (Grant and Curthoys, 2017). Evaluation of this range of NEL displacements utilizing the model, indicates that the higher this NEL stimulus displacement the higher the threshold maximum displacement value. The ratio of maximum threshold displacement to maximum stimulus displacement remains constant at 1.4 over the frequency ranges examined here.

### Striolar Hair Cell Bundle Stimulus

**FIGURE 15:**
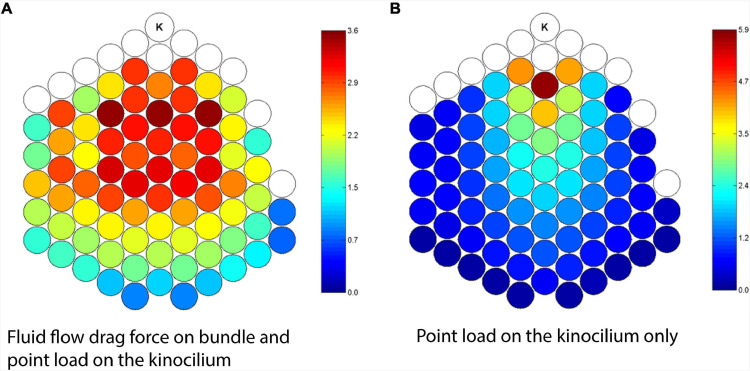
Striolar Type I hair cell bundle viewed from above with different deflection loadings. In these diagrams, each circle represents a stereocilium or kinocilium (designated K), with stereocilia without a tip-link are in white. Tip-link tension color-scales are shown to the right of each figure in terms of pN (piconewtons) force above a resting tension of 20 pN. The resting tension is produced by the molecular motors that hold the tension at a value just below channel open probability with the bundle at rest. Any stereocilium with an increased tip-link tension of 1.0 pN above resting is considered at 50% open probability. The Bundle to the left **(A)** is loaded with both fluid flow producing a drag force on the bundle and a point load on top of the kinocilium. All tip-links in this bundle are well above the 50% open probability tension and are considered open. The bundle to the right **(B)** is only loaded with a point force load on the kinocilium top, and with no fluid flow drag force loading. A large fraction of the tip-links around the periphery are well below the 50% open probability range. Those at the bottom are not tensioned at all. Only the central portion of the bundle has well opened tip-link channels. Tip-link tensions are shown after a full dynamic loading time period of 1 ms (Nam et al., 2005).

Inner hair cells in hearing are deflected by the relative motion between their base and the surrounding endolymph fluid (Freeman and Weiss, 1990a,b). In a similar manner, type I hair bundles in the striolar region of the utricle are also deflected by the relative motion between their base and the surrounding fluid, as well as being somewhat or loosely attached to the OL. The term relative motion here refers to the fact that in both cases it is the base that is in motion in relation to an inertial reference. For the otolith, in accelerometer mode, not only is neuroepithelial layer (NEL) in motion, the OL is also in motion lagging behind due to its inertia, resulting in relative displacement between the two layers. This is also true for the otolith in the seismometer mode, where it is the NEL that is in motion with the OL remaining at rest or only slightly in motion, again due to its inertia. In all cases, auditory inner hair cells, utricle striolar hair cells in acceleration mode and seismometer mode, it is the relative motion of endolymph fluid flowing over hair cell bundles, producing a drag force on the bundle causing them to deflect and so activating the tip-links as shown in Figure 15. This has been termed the shear force due to viscous coupling or viscous drag.

Utricle striolar bundles, are structurally stiff and are large bundles with large numbers of stereocilia. These bundles appear to be weakly attached to the OL, and they are likely deflected by the relative motion of endolymph flow drag. Finite element modeling shows that with only attachment to the OL, only a central section of these bundles activated their tip-link channels. With the relative motion and relative flow of endolymph over the bundle, all the tip-link channels are opened, providing a robust stimulus to these cells (Nam et al., 2005). The two extremes of stimulus are shown schematically in Figure 15: A. both OL displacement and fluid forcing and B. OL displacement alone.

The next steps in the unfolding VEMPs story will hopefully elaborate on the different tip-link patterns for different stimuli.

## Summary and Conclusion

We have given a broad overview of the clinical vestibular (otolithic) response – the vestibular evoked myogenic potential (VEMP) – to sound or vibration. We summarized the neural projections responsible for VEMPs and the neurophysiological results from *in vivo* extracellular recordings of single mammalian primary otolithic afferent neurons which provide the evidence that the VEMP is generated from a small subset of otolithic afferents with irregular resting discharge originating from receptors at the striola of the otolithic maculae. These afferents have a sensitive response to sound and vibration and show precise phase locking. Other otolithic afferents with regular resting discharge originating from extrastriolar receptors, are not activated by sound and vibration even at high intensities but show sensitive response to low frequency linear acceleration (<50 Hz). So, the otolithic maculae, like the retina, have two co-existing afferent neural systems – the transient system from the striolar afferents and the sustained system from the extrastriolar afferents.

A major objective of this publication was to communicate the contribution of the high frequency seismometer mode of otolithic response for understanding the measurement of VEMPs. For this seismometer mode of otolithic responding we addressed its mechanical origin, neural pathway, high sensitivity, phase locking capability, and overall behavior and compared this mode to auditory transduction. The significance of this high frequency mode of vestibular operation was not addressed. In daily life it may operate in two ways:

(1) As an initial signal to alert and to prepare muscles for a coming signal for contraction. This process is utilized in order to speed up the neural reflex, minimizing response time from stimulus until a muscle corrective action through contraction is taken. This can be applied to not only the vestibulo-ocular response during rapid head movements, but also is used for rapid response of the musculoskeletal system in situations such as slips or potential falls.
(2) It may also be used as a sound receptor for signals in the 0.5–3 kHz range. More than likely, this is an evolutionary remnant from fish which used this system for sea water hearing. However, it is still functioning in humans, and more than likely mammals have evolved to use this capability for rapid locomotion response to falls or other activities that require fast muscular response and rapid visual acuity in time of rapid head motion. The vestibulo ocular response is recognized as probably the fastest human reflex.

Section “ELECTROPHYSIOLOGY” addresses the main question – how is it that otolithic receptor cells are activated by sound and vibration. We summarize recent electrophysiological recordings from *in vivo* recording of mammalian utricular receptors showing that during stimulation by sound or vibration the otolithic maculae have similar electrophysiological potentials as cochlear receptors indicating receptor activation – both show microphonics, summating potentials, and gross evoked neural potentials. These electrophysiological results show that the VEMP can be thought of as the clinical myogenic correlate of the vestibular short-latency evoked potential (the VsEP) which is the vestibular correlate of wave I of the ABR. In these experiments any cochlear contribution was eliminated because the cochlea had been ablated so the potentials were purely vestibular. The similarity between the cochlear and vestibular system is to be expected since otolithic receptors are the evolutionary precursors of cochlear receptors and afferents (Straka et al., 2014).

“MECHANICS OF OTOLITHS IN VEMP TESTING” section uses mathematical modeling to show how the otolithic maculae with its layer of dense otoconia can allow the generation of hair cell and afferent response to such high frequencies. The modeling shows that the physical characteristics of the otolithic macula allow the system to respond to both very low frequency stimuli (accelerometer mode) and very high frequency stimuli (seismometer mode). The high frequency response has been especially puzzling because of the dense layer of otoconia overlying the receptors. But receptors at the striola are likely activated by fluid displacement around the hair bundle stereocilia which are tenuously attached to the otoconial membrane. The modeling not only predicts that the peak macula frequency response occurs at about 500 Hz but also that the peak frequency response shows an upward shift (to 1000 Hz) in patients with Menière’s Disease.

In summary we have shown the receptor, the neurophysiological and the mechanical basis of VEMPs to sound and vibration and the similarities between vestibular and cochlear receptor electrophysiology. Of course, major questions remain: (1) a detailed time series analysis of the macula movement which will explain the extremely fast response of primary otolithic afferents; and (2) what causes the differences between sound and vibration in clinical responses; and (3) what is the exact stereociliary mechanism triggering the intracellular receptor changes in striolar receptors.

## Author Contributions

IC wrote the manuscript. JG wrote the section “Mechanics of Otoliths in VEMP Testing” about the accelerometer-seismometer model evidence. CP and DB wrote the section titled “Electrophysiology” about *in vivo* evoked potentials such as the microphonic and compound action potential. IC and LF wrote the section about clinical evidence. All authors reviewed and approved the text of the final manuscript.

## Conflict of Interest

The authors declare that the research was conducted in the absence of any commercial or financial relationships that could be construed as a potential conflict of interest.

## Publisher’s Note

All claims expressed in this article are solely those of the authors and do not necessarily represent those of their affiliated organizations, or those of the publisher, the editors and the reviewers. Any product that may be evaluated in this article, or claim that may be made by its manufacturer, is not guaranteed or endorsed by the publisher.

## Abbreviations

ACS: air conducted sound
ANN: auditory nerve neurophonic
ABR: auditory brainstem response
BCV: bone-conducted vibration
CF: characteristic frequency
CFL: column filament layer
CM: cochlear microphonic
CAP: compound action potential
ECochG: electrocochleography
GL: gel layer
HC: hair cell
HCB: hair cell bundles
IHC: inner hair cell
IO: inferior oblique eye muscle
K+: potassium ion
MD: Menière’s Disease
MET: mechanoelectrical transduction channels
NEL: neuroepithelial layer
OAE: otoacoustic emission
OHC: outer hair cell
OL: otoconial layer
OM: otolithic membrane
pN: picoNewtons
RMP: resting membrane potential
SCM: sternocleidomastoid muscle
SL: shear layer
SCD: semicircular canal dehiscence
SP: summating potential
UDNF: undamped natural frequency
UM: utricular microphonic
VM: vestibular microphonic
VEMP: vestibular evoked myogenic potential.
oVEMP: ocular vestibular evoked myogenic potential
cVEMP: cervical vestibular evoked myogenic potential
VsEP: vestibular evoked potential.
